# Assessing Regional-Scale Impacts of Short Rotation Coppices on Ecosystem Services by Modeling Land-Use Decisions

**DOI:** 10.1371/journal.pone.0153862

**Published:** 2016-04-15

**Authors:** Jule Schulze, Karin Frank, Joerg A. Priess, Markus A. Meyer

**Affiliations:** 1 Department Ecological Modelling, UFZ – Helmholtz Centre for Environmental Research, Leipzig, Germany; 2 University of Osnabrück, Institute for Environmental System Research, Osnabrück, Germany; 3 Department Computational Landscape Ecology, UFZ – Helmholtz Centre for Environmental Research, Leipzig, Germany; 4 Department of Geography, University of Munich (LMU), Munich, Germany; DOE Pacific Northwest National Laboratory, UNITED STATES

## Abstract

Meeting the world’s growing energy demand through bioenergy production involves extensive land-use change which could have severe environmental and social impacts. Second generation bioenergy feedstocks offer a possible solution to this problem. They have the potential to reduce land-use conflicts between food and bioenergy production as they can be grown on low quality land not suitable for food production. However, a comprehensive impact assessment that considers multiple ecosystem services (ESS) and biodiversity is needed to identify the environmentally best feedstock option, as trade-offs are inherent. In this study, we simulate the spatial distribution of short rotation coppices (SRCs) in the landscape of the Mulde watershed in Central Germany by modeling profit-maximizing farmers under different economic and policy-driven scenarios using a spatially explicit economic simulation model. This allows to derive general insights and a mechanistic understanding of regional-scale impacts on multiple ESS in the absence of large-scale implementation. The modeled distribution of SRCs, required to meet the regional demand of combined heat and power (CHP) plants for solid biomass, had little or no effect on the provided ESS. In the policy-driven scenario, placing SRCs on low or high quality soils to provide ecological focus areas, as required within the Common Agricultural Policy in the EU, had little effect on ESS. Only a substantial increase in the SRC production area, beyond the regional demand of CHP plants, had a relevant effect, namely a negative impact on food production as well as a positive impact on biodiversity and regulating ESS. Beneficial impacts occurred for single ESS. However, the number of sites with balanced ESS supply hardly increased due to larger shares of SRCs in the landscape. Regression analyses showed that the occurrence of sites with balanced ESS supply was more strongly driven by biophysical factors than by the SRC share in the landscape. This indicates that SRCs negligibly affect trade-offs between individual ESS. Coupling spatially explicit economic simulation models with environmental and ESS assessment models can contribute to a comprehensive impact assessment of bioenergy feedstocks that have not yet been planted.

## Introduction

The world’s energy demand is continuously growing [1, 2]. Meeting this demand through bioenergy production involves extensive land-use change which could have serious environmental and food security implications [3]. For example, bioenergy expansion could negatively affect biodiversity [4] or cause indirect land-use change [5]. One possible solution to this problem are second generation (2G) bioenergy feedstocks: “perennial, ligno-cellulosic feedstocks that are non-food crops” [6] promoted by the EU through the Renewable Energy Directive (EU RED) [7]. They may reduce conflicts with food production as they can be grown on low quality land that is unsuitable for food production [8]. However, science-based safeguards need to be put in place to ensure that the best feedstocks for avoiding negative social and environmental impacts are exploited for bioenergy production [9]. In the process, trade-offs between provisioning and regulating ecosystem services (ESS) (i.e., human benefits from the ecosystem [10]) need to be evaluated [11–13]. Therefore, a comprehensive impact assessment of 2G feedstocks considering multiple ESS is required. In temperate climate zones, short rotation coppices (SRCs) are prominently discussed 2G feedstocks [14].

SRCs are fast-growing trees, in the EU mostly poplar and willow species, which are partly commercially grown as perennial energy crops on agricultural land [15]. Plantations are harvested every 3–9 years and afterwards stump sprouting takes place. After several of these rotations, the land is re-cultivated. SRCs may fulfill multiple bioeconomic purposes: they serve as a material source and feedstock for heat and electricity generation. At the same time, SRCs are thought to increase biodiversity [7, 16, 17] and positively affect soil and water quality [18, 19]. Furthermore, under the reformed Common Agricultural Policy (CAP) (2014–2020) farmers receiving subsidy payments are obliged to reserve at least 5% of their arable land for ecological focus areas (EFAs). SRCs are regarded as EFAs due to their beneficial impacts on the environment [20]. Despite the expected environmental benefits, currently only approximately 6500 ha SRCs are established in Germany [21]. However, Kraxner *et al*. [22] project a worldwide increase in SRC plantations to 190–250 million ha by 2050.

Several studies have modeled the potential supply of perennial energy crops and the accompanying impacts (e.g., [19], Meehan *et al*. [23], Aust *et al*. [24], Tölle *et al*. [25]). Thereby, it is crucial to consider the spatial configuration of energy crops in ESS assessments [26]. Holland *et al*. [7] and Milner *et al*. [6] emphasize the need to conduct assessments on commercial scale feedstock production systems. SRCs are currently seldom implemented and therefore empirical data on the spatial allocation of SRCs is missing. Models simulating upscaling processes therefore use heuristics to allocate new land-use options such as SRCs in the landscape. Meehan *et al*. [23], for example, replace annual with perennial energy crops. Tölle *et al*. [25] allocate SRCs on land with suitable cultivation conditions (e.g., sufficient available soil water capacity). Scenarios that reflect farmers’ decisions within the existing and potential future political framework are needed. Based on these scenarios, more realistic spatial allocations of SRCs can be determined. Several studies have shown that it is important to include human decision-making in models [27, 28].

In this study, we use a spatially explicit economic simulation model to simulate farmers’ decisions about their agricultural activity, such as the cultivation of a certain crop (SRC or conventional annual crops). While the farmers decide according to their individual cultivation conditions (biophysical cultivation conditions, transport costs), endogenous markets mediate interactions among them. With this model, we investigate four economic scenarios differing in the demand for SRC products. These scenarios reflect the maximum range of outcomes, which thereby more likely comprise the actual future of SRC deployment.

Embedded in the recent discussion on “Greening” in Germany [29], we also assess two policy scenarios where a certain share of the landscape is used for cultivating SRCs to fulfill the CAP requirements. We apply the model to the Mulde watershed in Central Germany. At this regionals scale, we assess the impact of SRC expansion on crop yields, carbon storage, nutrient and sediment retention, as well as biodiversity by using the environmental and ESS assessment models InVEST and GLOBIO. We cover these local/regional ESS as those are seldom included in common environmental assessments of bioenergy feedstock production, e.g., LCAs [30, 31]. Overall, we simulate land-use maps of SRC production and quantify ESS synergies and trade-offs resulting under different economic and policy-driven scenarios at the regional scale. The study aim is to derive general insights and mechanistic understanding of ecosystem service impacts of large-scale SRC deployment and to visualize some potential futures for SRC deployment.

## Materials and Methods

In this study, we combine a spatially explicit economic simulation model with environmental and ESS assessment models to assess the impact of land-use decisions on provisioning and regulating ESS and biodiversity. In the first part of the analysis, we use the economic simulation model to generate land-use patterns for a specific case study under different economic scenarios. Thereby, we assess a range of outcomes, which more likely comprise the actual future of SRC deployment. In the second part of the analysis, we use environmental and ESS assessment models InVEST and GLOBIO to assess ESS supply in a spatially explicit way. Here, we apply this modeling framework to the expansion of SRCs as 2G feedstock in the Mulde watershed in Central Germany.

### Study site

**Fig 1:**
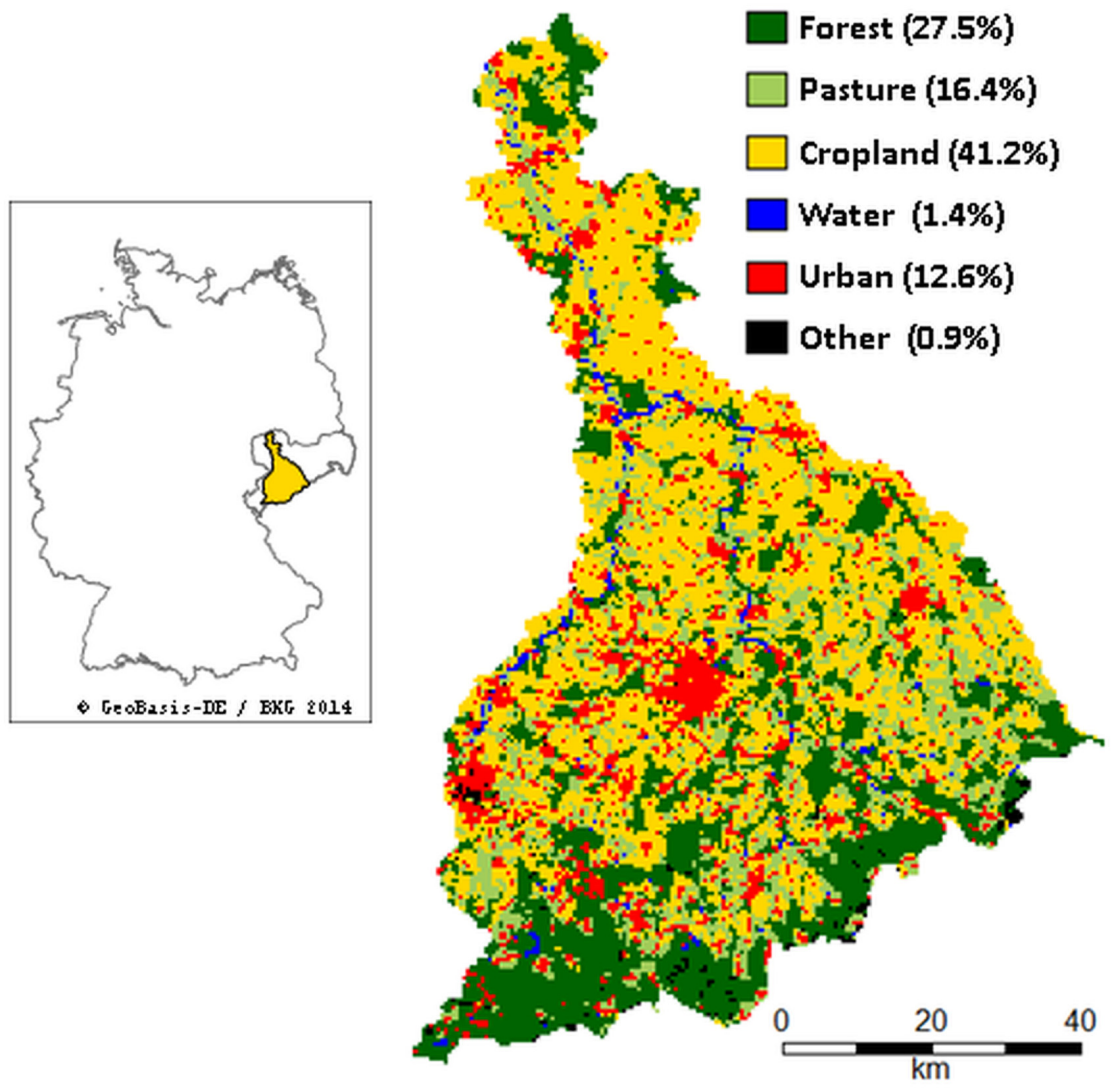
Land-use/land-cover in the Mulde watershed and its location in Germany [36–38].

The study area is part of the Mulde watershed, which is mostly located in the German federal state of Saxony (see Fig 1), covering an area of about 5,791 km². The Mulde is a tributary of the Elbe river formed by its headwaters Zwickauer Mulde and Freiberger Mulde, which have their source in the Ore Mountains. Its altitude ranges from 70 to 1214 m. We modeled SRC deployment and ESS for the reference year 2006, for which major land-use/land-cover (LU/LC) data is available. Climatically, the precipitation in 2006 in the Mulde watershed with its humid continental climate (834.6 mm, SD: 180.6 mm) was 9% lower than the normal climate conditions for the period 1991 to 2011 [32]; the minimum and maximum average temperatures in 2006 in the Mulde watershed (T_min_: 4.5°C, SD: 0.8°C, T_max_: 13.1°C, SD: 1.4°C) deviated less than 1°C from normal climate conditions for the period 1991 to 2011 [32]. The amplitude of precipitation ranged between 500 mm and 1290 mm in 2006. The loess soils in the lowlands are dominated by crop production, whereas the Ore Mountains are dominated by forestry [33]. Winter wheat (24%), winter rapeseed (18%), and winter barley (12%) dominated the cropland in 2006. Currently, SRCs account for only 0.03% of the agricultural land in Saxony [34]. There are only a small number of SRC sites in the Mulde watershed, most of which are trial sites. In contrast, there are about 36 combined heat and power (CHP) plants in Saxony [35] that may use SRC products. Fifteen of these CHP plants are located in the Mulde watershed.

### Economic simulation model

The spatially explicit model depicts land-use decisions of multiple profit-maximizing farmers who interact via an economic market. The model is an extended version of the model described in Weise [39]. Here, we present a short description of the model; the full model description using the ODD+D protocol [40] can be found in the S1 Text. The ODD+D extends the widely used Overview, Design Concepts and Detail (ODD) protocol [41, 42] by including the description of human decision-making.

The landscape of the Mulde watershed is subdivided into pixels. Each cropland pixel [37, 38] was assigned to an individual land user (agent). The underlying landscape consisted of a soil quality layer [43] and the sites where consumers of SRC products, i.e., CHP plants, were located [35]. The agent cultivated land to generate income through the production of agricultural goods. The agent could choose between three different land-use options: SRCs, annual crops, or fallow land. Among these, the agent chose the land-use option that would yield the maximum net profit. The net profit for a land-use option was given by the difference between revenue and costs. The revenue was influenced by market prices and the site-specific yield, while the costs were incorporated via production and transportation costs. The yield was determined by biophysical site-conditions, i.e., soil quality, while transportation costs of SRC products depended on the distance to CHP plants. Although traditional and self-interested profit maximization (i.e., the rationale of homo economicus) is widely accepted as a decision criterion in economics, non-commercial factors are also believed to influence agricultural decisions [44]. However, Brown *et al*. [45] show with a survey in the UK that economic factors are of primary importance. Non-economic factors such as the willingness to reduce GHG emissions are less important; they only slightly influence decisions to cultivate bioenergy crops. Therefore, we see this simplification as appropriate for our model design. For SRC practice in Germany, farmers are provided with advisory material (e.g., the manual of Skodawessely *et al*. [46] or the profitability calculator provided by the research project AGROWOOD [47]). To reflect the situation in practice, we adapted the profitability calculation suggested therein as a decision criterion.

Market prices for wood chips from SRCs and for annual agricultural products were given by the balance of exogenously given demand and the endogenously resulting supply that was determined by the agents’ decisions. Agents interacted indirectly via the resulting market price, i.e., agents reacted to market prices and these market price were determined by the decisions of all agents. This price formation on the market is in line with standard economic theory (e.g., equilibrium concept; cf. [48, 49]). Thereby, we incorporated the critical market feedback of supply decisions that result in price changes which influence again supply decisions (as also used by Lawler *et al*. [50]). We assumed that the market price was equivalent across all CHP plants and was determined by the joint supply from all agents (termed “regional price”). Besides utilizing SRC products, CHP plants were assumed to distribute resources: plants with a high supply (higher as their own capacity) might transport and sell to another plant or other customers. However, we tested this scenario against a second scenario in which the market price was formed for each of the CHP plants and was determined by the amount of SRC that was sold to a specific plant (termed “local price”). Here, market prices might vary between plants. The two investigated price scenarios represent two extremes at opposite poles of a spectrum. Simulated land-use patterns and the regional ESS showed to be mostly equivalent across both price scenarios, cf. Table A and B in S1 File. Hence, it is unlikely that the actual land-use patterns and ESS supply differ substantially from these two conceptual extremes. Here, we focus on the “regional price” scenario and show results for the “local price” scenario in the appendix.

The majority of parameters in the economic model were based on the literature (for details see full model description in the S1 Text). The demand for annual agricultural crops was calibrated using the LU/LC data for the Mulde watershed. This demand was set by aligning the initial shares of land-use options under the baseline scenario with the empirical ones in the Mulde watershed.

### Scenarios for SRC development

For the standard scenario, we assumed that the CHP plants currently present in the Mulde watershed were the only consumers of wood chips and that their biomass demand was fully met by wood chips from SRCs. We calculated the share of SRCs needed to meet the demand of existing CHP plants in the Mulde region by using the reference values from the FNR [51] as a basis. Using this information, we parameterized the demand for SRC products in the economic simulation model so that the required SRC share was provided in the modeled region.

Increasing global demand for wood combined with limited forest resources will most likely increase prices for wood in the future [52]. Therefore, we assessed the impact of an increasing demand for wood chips by comparing the standard scenario with three further scenarios (medium, high and very high demand). In these additional scenarios, we did not spatially allocate additional CHP plants in the landscape because the regional energy and heat demand is unlikely to increase further. We rather expected that other than energetic uses in CHP plants (e.g., material use) increased the regional demand for wood resources [53]. This is in line with [54], who identified a likely solid biomass supply deficit in Germany that could be partly filled with SRCs. To depict this in the economic model, we increased the demand for SRC products over the different scenarios and modeled SRC production for other regional uses. Thereby, we assumed that the existing CHP plants acted as trading centers and that farmers delivered SRC products to the already established infrastructures. By considering this range of demand for SRC products, we aimed at investigating the maximum variation in biodiversity and ESS supply that might be caused by expanding SRC production. The actual outcomes will then likely be bounded by this range.

In addition to the economic scenarios, we included two policy scenarios. Embedded in the recent discussion on “Greening” in Germany [29], we assumed that 16.67% of the entire arable land is used for cultivating SRCs. Under the recent CAP reform, farmers need to implement EFAs on 5% of their land to receive payments. One of many options is to plant SRCs. However, SRCs are weighted less (weighting factor: 0.3) than set-aside land (weighting factor: 1). Hence, 16.67% (= 5%/0.3) of land needs to be used for SRC cultivation. Again, we investigated two extremes to gain understanding on the range of possible outcomes of this policy intervention. In the first policy scenario, we assumed that SRCs would be allocated to land with low soil quality for economic reasons. We assumed potential deficiencies of this policy measure (i.e., EFAs) due to the freedom of location choice for farmers. Therefore, we compared this scenario to a second policy scenario where the best 16.67% of the entire arable land with respect to soil quality would be converted to SRCs to analyze potential ESS impacts.

### Ecosystem services and biodiversity

#### Provisioning ecosystem services

For all scenarios, we calculated the crop and SRC yields. For crop production, we spatially downscaled the average yield per ha at the district level [55, 56]. We considered the impact of soil and climatic heterogeneity on yields by calculating the arithmetic mean of the agricultural yield potential for each district [57]. We assigned each pixel the yield available at district level and raised or lowered this value depending on the actual agricultural yield potential of the pixel relative to the district arithmetic mean.

We selected the SRC species poplar due to the dry climate in the agriculturally dominated lowlands. To model a spatially explicit KUP yield, we used the regression model developed in Saxony by Ali [58]: (1) $Yield=a_{4}{\left(a_{1}*C+a_{2}*P*SQI+a_{3}*\frac{T}{AWC}\right)}^{a_{5}}$ with (2) *a*_4_ = −1.13 * 10^−9^ * *N*^2^ + 2.54 * 10^−5^ * *N* + 0.028 and (3) *a*_5_ = 3.41 * 10^−9^ * *N*^2^−5.01 * 10^−5^ * *N* + 2.614 where *C* is the rotation cycle, *P* the sum of precipitation for the months May and June, *SQI* the soil quality index, *T* the average temperature for the months April until July, *AWC* the available water holding capacity, *N* the planting density and *a*_*1*_ up to *a*_*5*_ are species-specific parameters. Based on the existing practice in Saxony, we assumed the use of the most common poplar clone Max. with the parameters and datasets given in Table 1.

**Table 1:** Parameter and datasets used to calculate yield of the poplar clone Max.

	Item	Value	References
N	Planting density [*n* ha^-1^]	9446	TU Dresden/AgroForNet [34]
C	Rotation cycle [a^-1^]	5.5	TU Dresden/AgroForNet [34]
a_1_		1.569	Ali [58]
a_2_		0.0004	Ali [58]
a_3_		-23.198	Ali [58]
P	Precipitation (sum May-June) [mm]		Jäckel *et al*. [32]
T	Average temperature April-July [°C]		Jäckel *et al*. [32]
SQI	Soil quality index		LfULG [43]
AWC	Available water holding capacity [mm]		LfULG [43]

#### Regulating ecosystem services and biodiversity

We used InVEST (Integrated Valuation of Environmental Services and Tradeoffs) to calculate different regulating ESS. InVEST uses ecological production functions to simulate the provision of ESS under different scenarios. First, we calculated the amount of carbon stored according to the IPCC guidelines [59], supported by InVEST [60, 61], with the data indicated in Table 2 for 2006. Second, we modeled the phosphorous (P) export and retention with InVEST. Based on the runoff, the P inputs were routed through the watershed. The retention largely depends on the topography and vegetative cover. Third, we modeled the amount of retained sediment with InVEST based on the universal soil loss equation [62, 63]. The baseline scenario for P and sediment retention was validated in Meyer *et al*. [64].

**Table 2:** Data items for carbon storage (No. 2, 16), P retention and export (No. 1–7, 10–12), sediment retention and export (No. 1–3, 8–9, 13–15 and for biodiversity (No. 2, 17–21); we refined the default parameter of InVEST with the indicated sources (No. 10–15); methodological sources are equally included.

	Input datasets	References
1	DEM (3 arc-seconds) [m]	Lehner *et al*. [83]
2	LU/LC	European Environment Agency (EEA) [36], Wochele *et al*. [37], Wochele-Marx *et al*. [38]
3	Potential Natural Vegetation	LfULG [84]
4	Reference Evapotranspiration (10 arc-min) [mm a^-1^]	FAO Geonetwork [85]
5	Precipitation [mm a^-1^]	Jäckel *et al*. [32]
6	Depth to any soil restrictive layer [mm]	Panagos *et al*. [86], LfULG [43]
7	Available water holding capacity [cm cm^-1^]	Panagos *et al*. [86], LfULG [43]
8	Erosivity (R) [MJ mm ha^-1^ h^-1^ a^-1^]	Bräunig [87]
9	Erodibility (K) [t ha h ha^-1^ MJ^-1^ mm^-1^]	LfULG [43], Bischoff [88]
10	Rooting depth [mm]	
11	P export [kg ha^-1^ a^-1^]	Reckhow *et al*. [89]
12	P retention efficiencies [*%*]	
13	cover-management factor (C)	
14	Support practice factor (P)	SMUL [90]
15	Vegetation sediment retention efficiency [*%*]	
16	Carbon pools [t ha^-1^]	Fraver *et al*. [91], Bohn *et al*. [92], Müller-Using and Bartsch [93], Wördehoff *et al*. [94], Polley and Henning [95], Strogies and Gniffke [96], BGR [97]
17	Population density [*n* km^-2^]	Priess [98], Priess *et al*. [99]
18	Street and railway map	BKG [100]
19	N deposition	Builtjes *et al*. [101]
20	Critical N loads	Builtjes *et al*. [101]
21	Terrestrial ecoregions	Olson *et al*. [102]

Furthermore, we assessed impacts on biodiversity with GLOBIO as described in Alkemade *et al*. [65]. We modeled the impact of major drivers of biodiversity loss (i.e., land-use change, habitat fragmentation, population density, infrastructure expansion and atmospheric N deposition) on the mean species abundance (MSA) existing in undisturbed ecosystems. A completely undisturbed ecosystem would have an MSA of 1, the lowest MSA is 0; SRCs have a value of 0.2.

#### SRC impacts on regulating ESS bundles

We used cluster analysis to identify ESS bundles based on methods described in Mouchet *et al*. [66]. ESS bundles are described as “sets of services that appear together repeatedly” by Raudsepp-Hearne *et al*. [12, p. 5242]. Pixels in our landscape were clustered into bundles of similar ESS supply and the frequency of each bundle over the entire landscape was recorded. We identified ESS bundles by K means and displayed them as starplots in R [67].

In a second step, we applied a binomial logistic regression model. This approach gives insights on the factors that distinguish the occurrence of different bundles. In the regression model, we tested indicators of landscape composition and naturalness, soil, topography, and climate, see Table A in S2 File. We calculated landscape composition and naturalness indicators in a moving window approach for a buffer radius of 5 km, which was ten times the LU/LC pixel size. We removed variables with variance inflation factors >10 to reduce multicollinearity. Next, we removed non-significant explanatory variables in a backward stepwise manner based on the Akaike information criterion. Next, the significance of the final model was tested against a null model using a likelihood ratio test. To assess the spatial autocorrelation of the final model, we added geographic coordinates and tested for significant difference towards the final model without geographic coordinates with the likelihood ratio test.

## Results

### SRC distribution and associated ecosystem services impacts under economic and policy-driven scenarios

**Fig 2:**
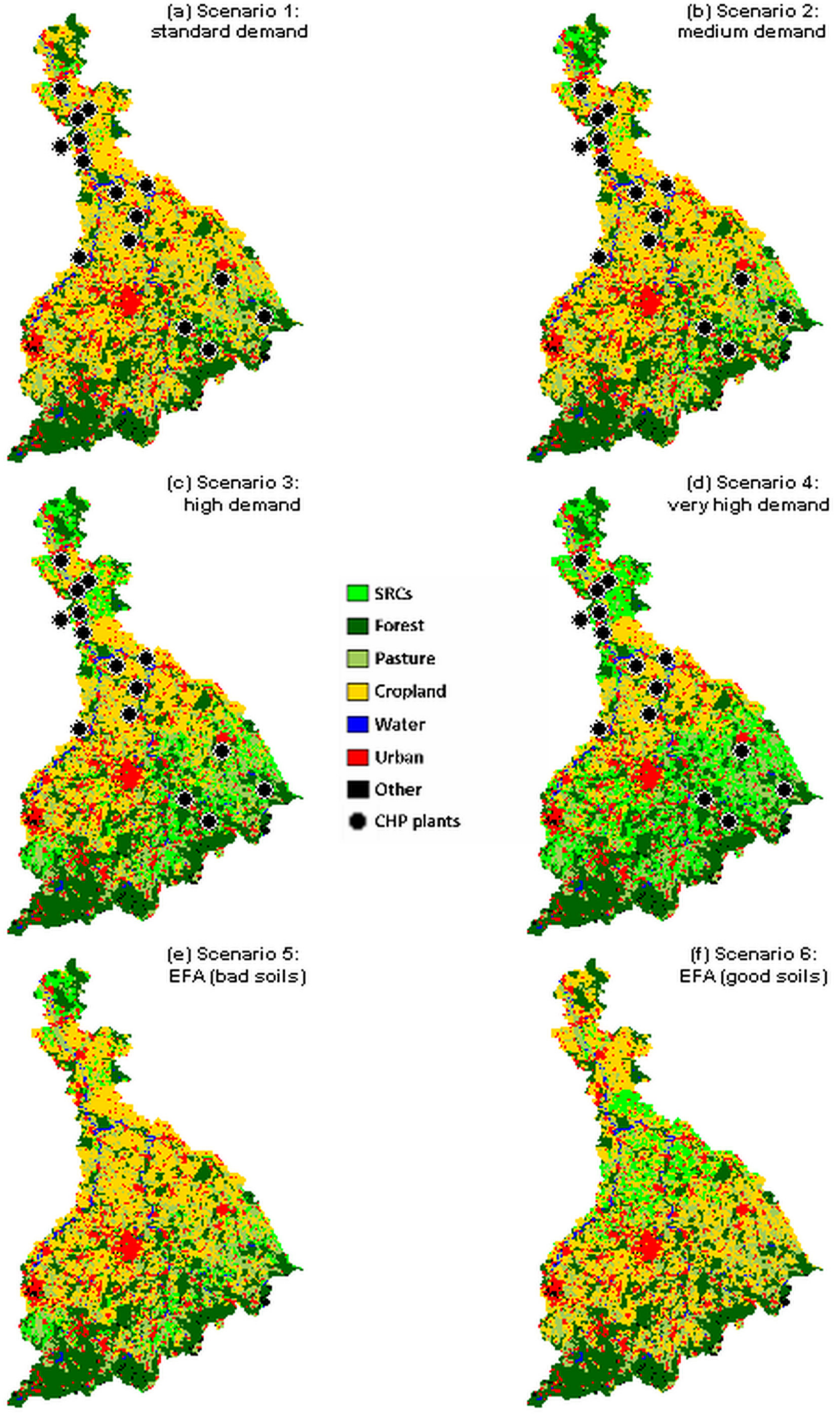
Deployment of SRCs in the Mulde watershed for four economic (1–4) and two policy-driven (5–6) scenarios. The economic scenarios are based on the economic simulation model. The policy scenarios reflect the potential deployment of SRCs to fulfill the requirements for EFAs (ecological focus areas). The dots indicate existing CHP plants [35].

Spatial distribution of SRCs depended on the economic and policy-driven scenarios (Fig 2). The share of SRCs differed substantially between the four economic scenarios (2%, 4%, 14%, and 24% of the total area for standard, medium, high, and very high demand, respectively). However, SRC distributions showed similar characteristics under the four scenarios: sites with high soil quality indices showed hardly any deployment. SRCs are only economically viable on inferior soils, where they can compete with annual crops. Distribution of SRCs under the first policy-driven scenario, where 16.67% of agricultural land with the lowest soil quality indices was converted to SRC cultivation (see scenario 5), was largely similar to the economic scenario with high demand.

**Fig 3:**
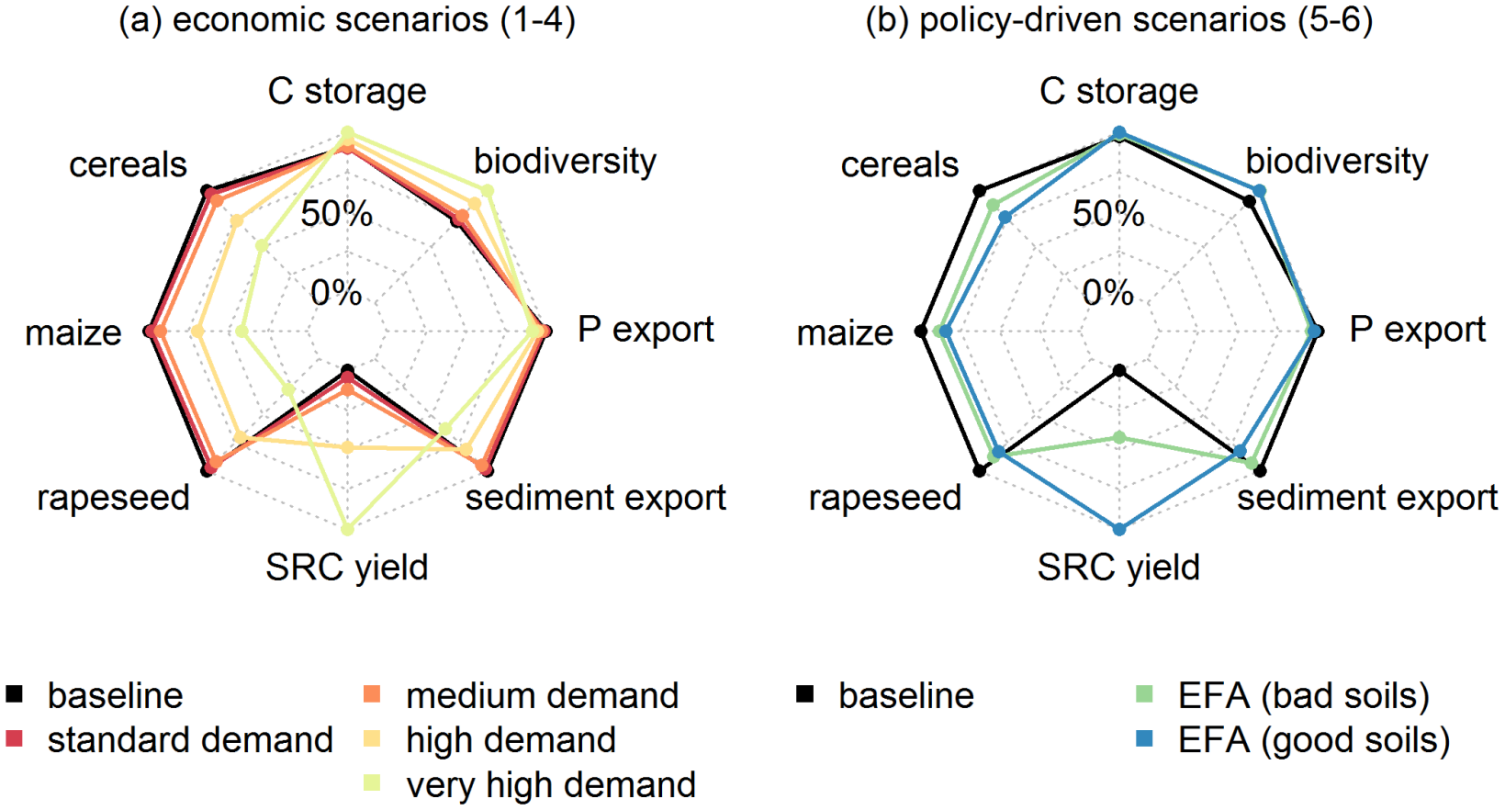
Trade-offs between provisioning and regulating ESS in (a) the economic and (b) the policy-driven scenarios, each set compared to the baseline scenario (black line). For each ESS, the scenario values are normalized with respect to the maximum value obtained; in other words, the maximum value of all scenarios is set to 100% and differences of the remaining scenarios are given in percent of the maximum value. For most of the ESS, higher values imply a better performance; a lower value is only better for P and sediment export.

In general, economic and policy-driven scenarios affected provisioning and regulating services as well as biodiversity (Fig 3). Scenario 1 (standard demand) (i.e., demand from the currently existing CHP plants solely met by SRCs) did not have a substantial effect on the investigated ESS. Only a large increase in demand (scenarios 3 and 4) for woody products from SRCs revealed substantial trade-offs between the provision of annual agricultural products and SRC yields as well as regulating ESS and biodiversity. For example, in scenario 3, compared to the baseline scenario, SRC deployment on 14% of the study area synergistically increased biodiversity (+22%) and carbon storage (+5%) and reduced P (-5%) and sediment export (-19%) from a regional perspective.

Interestingly, the two policy-driven scenarios led to different trade-offs. SRCs placed on good soils positively affected SRC yield (403,000 t per year), carbon storage (+3%) and sediment export (-18%) at high costs of annual crops (ranging from -16% to -23% depending on the crop) (see scenario 6 in Table A and B in S1 File). In contrast, SRCs placed on bad soils less positively affected SRC yield (170,000 t per year); annual crops were less negatively affected (ranging from -12% to -13% depending on the crop). It also led to a slightly higher reduction in P export (+4%) and a less positive effect on sediment export (-7%) and carbon storage (+1%) (see scenario 5 in Table A and B in S1 File). Effects on biodiversity hardly differed between the two policy-driven scenarios (ranging from 10% to 11%).

### SRC impacts on regulating ESS bundles

**Fig 4:**
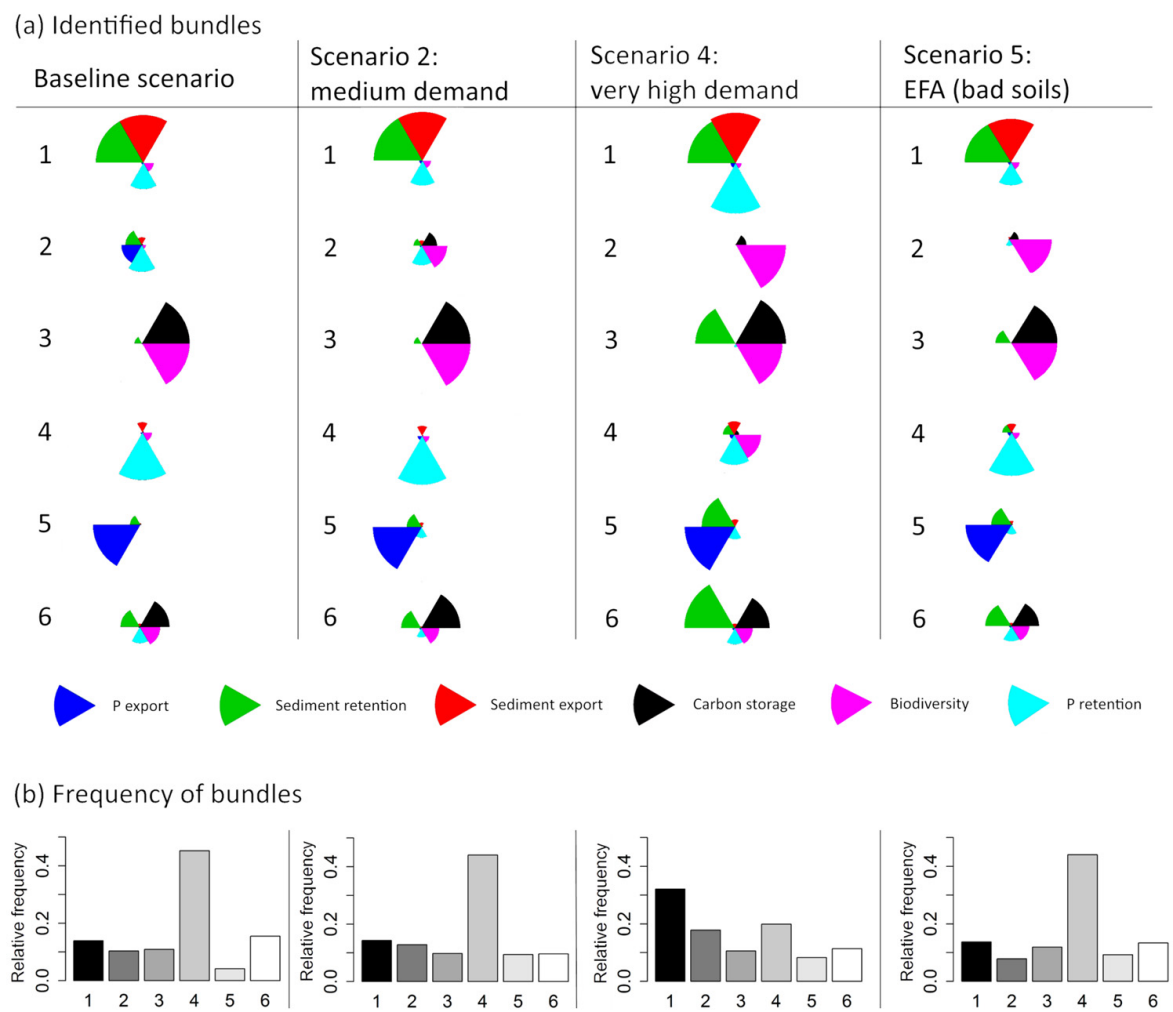
Identified ESS bundles (a) and their frequency (b) for the baseline scenario, two economic scenarios and a policy scenario. The highest arithmetic mean value for each ESS category is used as the maximum to scale the radar charts. The frequency of the ESS bundles is based on K means.

Most of the ESS bundles, i.e., locations with a set of comparable ESS values, prevailed independent of the share of SRCs in the landscape, which varied between the scenarios (see Fig 4a). Only ESS bundle 2 strongly differed between the baseline scenario, scenario 2 (medium demand) (balanced low-value bundle) vs. the more homogenous scenarios 4 (very high demand) and 5 (EFA (bad soils)) (high biodiversity bundle). Also bundle 4 in scenario 4 (biodiversity and P retention) slightly differed from the other scenarios. However, the frequency of the respective ESS bundles changed more strongly (Fig 4b). For example, bundle 2 with a high biodiversity value was much more frequent in scenario 4 than in scenario 5. Also bundle 1 (sediment and P retention and sediment export) was more frequent in scenario 4, but bundle 4 (P retention and biodiversity) was less frequent than in the other scenarios. This might be due to the fact that increased SRCs seemed to enhance the frequency of bundle 1 (sediment and P retention and sediment export). Especially P and sediment retention became more frequent in the economic scenario 4 (bundle 1) compared with the other scenarios; this partly reflects the dominance of SRCs in scenario 4 retaining more P and sediment from agriculture. The share of SRCs in the landscape made the beneficial balanced bundle 6 (sediment and P retention, carbon storage, and biodiversity) less frequent compared with the baseline scenario.

**Fig 5:**
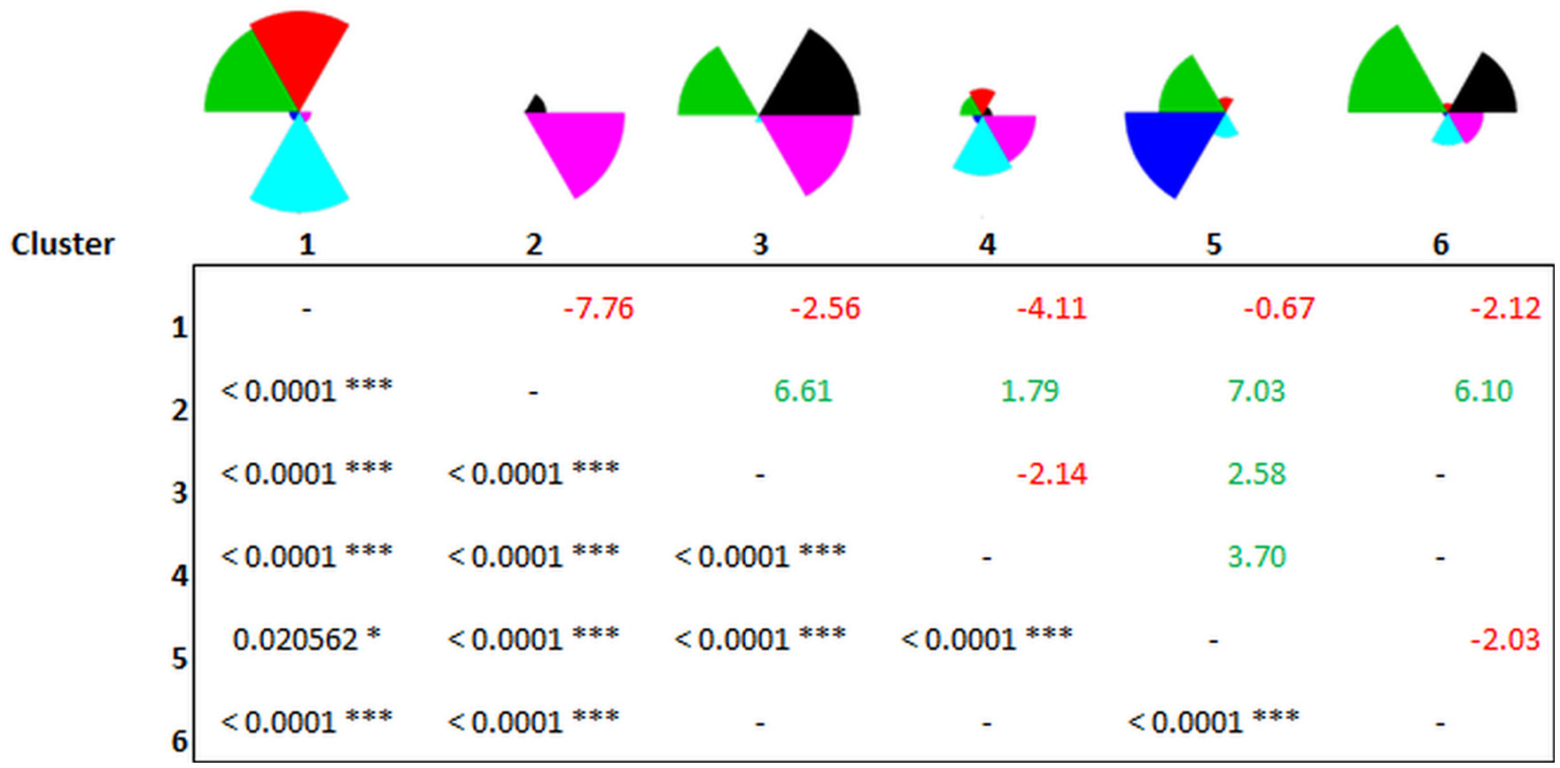
Percentage of SRC characterizing ESS bundles in scenario 4 (backward logistic regression). A positive value for the standardized β indicates that an explanatory variable is contributing to the cluster with the lower ordinal number; a negative value for the standardized β indicates that an explanatory variable is contributing to the cluster with the higher ordinal number. The entire regression results are listed in S1 File.

We applied a binomial logistic regression model to analyze the site-specific factors that determine the occurrence of different bundles in scenario 4 and to determine the role of SRCs relative to other factors. A higher share of SRCs surrounding a site enhanced the occurrence of bundles 2 and 4, see Fig 5. In that respect, a higher share of SRCs enhanced either (i) biodiversity or (ii) biodiversity, and P retention. A higher share of SRC weakened the occurrence of bundle 1 (sediment export and retention and P retention). However, the share of SRCs surrounding a site had little effect on the occurrence of the balanced bundle 6. In contrast, a higher slope and a higher available water holding capacity distinguished the balanced bundle 6 from the unbalanced bundle 5 with a dominance of P export (Table 3). Vice versa, a higher precipitation characterized bundle 5. The high explanatory power of the biophysical factors for the balanced ESS bundles and the rather low explanatory power of the share of SRCs showed that modifying landscape composition might be insufficient as exclusive measure (e.g., a high share of SRCs in the landscape to fulfill EFAs).

**Table 3:** Factors characterizing ESS cluster 5 and 6 for scenario 4 (backward logistic regression; p<0.001 (***), p<0.01 (**), p<0.05 (*), p<0.1(.)). A positive value for the standardized β indicates that an explanatory variable is contributing to cluster 5; a negative value for the standardized β indicates that an explanatory variable is contributing to cluster 6. The likelihood ratio test showed a significant difference when the final model was compared to a null model (χ2 = 1317.1, df = 13, p<2.2e-16). Comparing the final model with a model including x- and y-coordinates, the likelihood ratio test showed only a small difference (χ2 = 30.706, df = 3, p = 9.801e-7).

Explanatory variable	Stand. β	SE	z value	Pr(>|z|)	
(Intercept)	14.191	0.8487	16.72	< 0.0001	***
***Topography and soil parameters***					
Elevation [m]	-7.8992	0.795	-9.936	< 0.0001	***
Slope [%]	-12.1899	0.6934	-17.58	< 0.0001	***
Aspect [*°*]	0.6754	0.3015	2.24	0.0251	*
Curvature [*score*]	-4.9453	0.727	-6.802	<0.0001	***
Effective rooting depth [mm]	-2.525	0.5256	-4.804	<0.0001	***
Erodibility (K) [t ha h ha^-1^ MJ^-1^ mm^-1^]	-1.2373	0.5778	-2.141	0.0322	*
Available water holding capacity [cm cm^-1^]	-10.7915	1.5974	-6.756	<0.0001	***
***Climate***					
Precipitation [mm]	4.4494	0.5605	7.938	<0.0001	***
Reference Evapotranspiration [mm a^-1^]	-4.5254	0.5381	-8.41	< 0.0001	***
***Landscape composition***					
Forest, 5 km buffer [*%*]	-3.9183	0.559	-7.009	<0.0001	***
SRC, 5 km buffer [*%*]	-2.0335	0.3939	-5.162	<0.0001	***
Urban, 5 km buffer [*%*]	0.8486	0.4872	1.742	0.0815	.
Water, 5 km buffer [*%*]	-2.6205	0.4617	-5.676	<0.0001	***

## Discussion

### Ecosystem services and biodiversity under economic and policy-driven scenarios

A major aim of this study was to analyze the impact of SRCs on ESS and biodiversity and to identify synergies and trade-offs under different economic and policy-driven scenarios. By investigating different scenarios, we assessed a range of possible futures and thereby the maximum variation in outcomes; with the actual outcome being bounded by this range. SRCs are discussed as sustainable 2G feedstock for energy production. Our approach of placing SRCs in the landscape by modeling farmers’ decisions contributes to filling the gap in existing research which synthesizes mostly plot/field scale studies for ESS [6] and conceptually discusses but does not test the beneficial impact of SRCs on ESS at the regional scale [68]. Only few studies such as Fürst *et al*. [69] have assessed the impact of SRCs on multiple ESS and biodiversity at the regional scale, but without modeling farmers’ decision-making to assess commercial SRC deployment. Simulating farmers’ decisions and indirect market interactions allowed us to develop spatially explicit SRC distributions at a commercial scale, given the assumptions made in the economic simulation model. This enables an environmental and ESS impact assessment as requested by several authors for 2G feedstocks, e.g., Holland *et al*. [7]. We spatially explicitly model SRC allocations and impacts on multiple ESS, while existing SRC impact assessments, e.g., Milner *et al*. [6], focus on carbon storage.

In the investigated economic scenarios, farmers preferably cultivated SRC plantations in the southern and northern part of the Mulde watershed where sites with low-quality soils dominate. SRCs seem to compete with annual crops on these low quality soils. In that respect, we can confirm Hellmann and Verburg [70] who assume “[…] that it is unattractive to cultivate biofuel crops on locations with relatively very high yields of cereals and root crops due to economic competition” (p. 2414). Furthermore, our model results indicate that SRCs are established in the proximity of existing CHP plants. This is in line with Kocoloski *et al*. [71] and Vanloocke *et al*. [72] who state that 2G feedstocks are likely to be clustered around biorefineries. In scenario 1 (standard demand), we tested the impact of switching the current input of CHP plants in the Mulde watershed to wood chips from SRCs. We showed that the investigated provisioning and regulating ESS and biodiversity would be only slightly affected in scenario 1. In particular, food production will not be affected much. Only substantial promotion of SRCs would increase biodiversity and carbon sequestration as well as reduce sediment and P export. This is in line with Fürst *et al*. [69] who showed for a case study in Central Saxony, Germany, that a substantial increase of SRC production by up to 30% (depending on the region) would beneficially affect the provision of ESS and biodiversity. Meehan *et al*. [23] also showed a decrease in P export to surface water and an increase in carbon sequestration by switching from annual crops to perennial grasses. For the ESS assessment, we chose InVEST and GLOBIO as they allow us to model multiple regulating ESS with a high reliability relative to the effort involved, e.g., Meyer *et al*. [73]. Given that we conduct a relative comparison of the different scenarios, the possible inclusion of slight imprecisions, which are present to an equal extent in all scenarios, pose no major disadvantage.

Besides the economic scenarios, we assessed instruments in two policy scenarios for their impact on SRC deployment and ESS. We compared the more likely policy scenario 5 (“EFA (bad soils)”) with the rather hypothetical scenario 6 (“EFA (good soils)”). Both scenarios showed different impacts on the regarded ESS. In scenario 6, SRC yield, carbon storage, and sediment export are more positively affected at high costs of annual agricultural production. In contrast, scenario 5 is slightly more beneficial for P export while crop production is not tremendously decreased. Both scenarios have the same share of SRCs in the landscape, but with differing spatial distribution answering the research need raised by Holland *et al*. [7]. They ask whether the distribution of feedstocks might affect the provision of ESS. This underlines the importance of aiming at more realistic distributions of potential energy crop deployment (e.g., for example by explicitly modeling farmers’ decisions). In addition, it shows the potential to reflect on the current EU policy of coupling subsidy payments to the provision of EFAs. Our study reveals that the rules regarding the EFAs’ properties need to be specified depending on the environmental goal. For example, the same share of SRCs in the landscape led to significantly different reductions of sediment export compared to the baseline scenario (i.e., 7% (scenario 5) or 18% (scenario 6) (Table A in S1 File)). Therefore, the CAP should include the quality of the options available to fulfill EFAs as well as the biophysical site conditions. In addition to the share of SRCs in the landscape, biophysical factors were also important for increasing the frequency of balanced ESS bundles, see Table 3 and Tables B-O in S2 File. Therefore, a policy combining beneficial biophysical factors and land-use types might more strongly enhance ESS supply.

We clustered ESS bundles to analyze how the share of SRCs in the landscape affected the occurrence and frequency of the respective bundles. Thereby, we assessed occurring trade-offs between multiple ESS as well as biodiversity at the regional scale, which are inherent in the 2G feedstock expansion [11–13]. In that respect, our approach helps to balance competing services for deployment decisions as required by Holland *et al*. [7]. Comparing all scenarios with the baseline scenario, the different shares of SRCs did not enhance the balanced bundle 6. Comparing the different bundles in scenario 4 with a binomial logistic regression model, we showed that SRCs especially enhanced either (i) biodiversity (bundle 2) or (ii) biodiversity, and P retention (bundle 4), but did not enhance the balanced bundle 6. Such analysis on ESS bundles broadened the findings for single ESS from the synthesis by Holland *et al*. [7]. Balanced ESS bundles are unlikely to be obtained even with a high share of SRCs in the landscape. Their occurrence can be better explained by biophysical factors. Overall, the beneficial impact of SRCs on multiple ESS and biodiversity as discussed in several studies (e.g., Manning *et al*. [68] or Holland *et al*. [7]) was found to be rather low for the individual SRC plot and the regional scale.

### Methodological reflections and transferability of methods and results

In this study, we used a spatially explicit economic simulation model to simulate farmers’ decisions on their agricultural activity. While the farmers decide according to their individual conditions (yield and transport costs), endogenous markets mediate interactions among them. Agents react to market signals and in turn influence the market price which is given by the balance of demand and supply. Therefore, our approach builds on equilibrium theory as demand and supply meet in the equilibrium in the course of time. With the depiction of human decision-making depending on individual, heterogeneous cultivation conditions and the indirect market interaction, the presented modeling approach is close to the agent-based modeling (ABM) framework [74]. However, prominent ABM characteristics such as direct interactions between agents and heterogeneous agents’ decision-making are currently not incorporated into the model, so that it resembles a cellular automaton approach in some aspects.

The economic simulation model was developed to evaluate potential futures of SRC deployment when this production practice will reach its mature commercial phase. In this model, we assumed profit maximization as decision rule. Although this rational and self-interested decision-maker is widely accepted in economics, non-commercial factors are also believed to influence agricultural decisions [44]. In Germany, farmers’ interested in SRC practice are provided with advisory material (e.g., the manual of Skodawessely *et al*. [46]) or profitability calculators provided by projects (e.g., AGROWOOD [47]). Therefore, we implemented the profit maximization as determining decision criterion suggested there. We assumed farmers’ decision-making to be homogeneous because the structure of farms in the study region is very homogenous. Large cooperatives organized as legal entities succeeded the state-owned farms that existed during the GDR period and account for about three quarters of the entire agricultural area in eastern Germany [75]. In economic models, often budget constraints are incorporated to reflect limitations individuals face due to their available income. In this study, we did not include budget constraints as land rather than money is the critical input factor. The overall soil quality in the study region is rather high; therefore, farmers expectedly always cultivate their land if not hindered through policy (e.g., EFAs are mandatory as implemented in scenario 5 and 6) or other measures. Here, the question is rather, which demand level makes SRCs more profitable than annual crops. Therefore, the budget constraint is negligible.

Beyond modeling SRC deployment in the commercial phase, one could also model the current process of SRC deployment (e.g., to explain the slow deployment of SRCs). This would require additional factors such as practical challenges associated with SRCs and other new land-use options, which could be influential [76–78]. In this context, social influence from neighbors or decision-making concepts such as early or late adopters (as for example done in [79]) should be incorporated. Future research could adapt the model to address pre-mature commercial phases by including social influence or different decision types, so that the model more closely resembles an ABM framework.

Considering the discussed assumptions, the economic simulation model can be applied to different regions and policy settings. Within the assumed profit maximization, we focused on the major site-specific influence factors of cropping decisions (i.e., soil quality and distance to CHP plants). From the environmental perspective, this is an appropriate assumption for our study area as the entire arable land is located in a plain area with moderate variation in slope and available water holding capacity. In study regions, which also fulfill these characteristics, the economic simulation model can be reapplied to simulate SRC deployment if spatial data on soil quality or CHP plants’ distribution is available. If further biophysical factors, such as slope, are heterogeneous within the study region, these could be included in the economic simulation model.

However, without reapplying the ABM, the results of the ESS assessment should already be representative for similar German soil climate clusters (“Boden-Klima-Räume”), which comprise large areas of eastern Germany (Saxony and Thuringia (Saxon-Thuringian Hills and Upper Lusatia)) [80]. In addition, this region is one of Germany’s major crop production areas. With respect to land-use intensity, agricultural companies and field structures in this region remained mostly stable after the re-privatization of the Agricultural Production Cooperatives [75], which allow for comparable highly mechanized land management. Further environmental transfer of the results beyond Germany might be equally feasible with different approaches that control for environmental heterogeneity as developed in Meyer *et al*. [64].

From a policy perspective, increasing demand in the economic simulation model can resemble instruments like the promotional policies under the Renewable Energies Act in Germany and other national laws implementing the EU RED that affect the prices for woody biomass. Furthermore, these market-related changes may also be affected by emerging novel conversion technologies [54]. Beyond the EU RED, other environmental policies such as forest protection policies and reforestation initiatives may also affect biomass demand and cause regional shifts [81]. In that respect, our approach of coupling an economic model with ESS and environmental assessments may also be applied to assess the impact of changing supply and demand patterns in the forestry sector on regional ESS.

All assumptions discussed above influence our model results. In our study, however, the aim is not to predict land use in the Mulde watershed, but rather to derive some general insights and mechanistic understanding. Therefore, we follow a scenario approach. Scenarios hardly predict the exact land use that will occur, but rather show the range of potential futures (see for example Amer *et al*. [82]), i.e., in this study SRC deployment in the Mulde watershed. Thereby, we assess the maximum range of possible outcomes and expect that the actual outcome will lie within this range. Extensive empirical model validation on current SRC deployment is impossible; SRCs are seldom deployed which impedes an empirical validation of simulated land-use patterns with actual land-use patterns.

Coupling an economic simulation model with environmental assessment tools such as InVEST and GLOBIO offers several advantages over simpler suitability assessments (e.g., Meehan *et al*. [23] or Tölle *et al*. [25]). The latter often rely on predefined thresholds, but miss farmers’ decision-making in the site selection process. In our approach, modeling farmers’ decision-making determines the spatial deployment of SRCs. This makes it possible to apply the same methodology to different settings (e.g., other decision processes or different economic or policy scenarios) and to investigate how this changes the allocation of SRCs without predefined suitability rules. Suitability rules are unlikely to be transferable even at the national scale due to heterogeneous management and environmental conditions.

## Conclusions

In this study, we assessed how SRCs would affect multiple ESS and biodiversity under different economic and policy-driven scenarios in the Mulde watershed in Central Germany. We found that only a substantial increase in SRC production areas will considerably reduce food production and increase the provision of regulating services. However, there is hardly any increase in the number of sites with balanced ESS supply due to larger shares of SRCs in the landscape: SRCs do not significantly enhance the multifunctionality of the landscape. By modeling land-use decisions, we simulate a more realistic spatial distribution of bioenergy feedstocks under future scenarios. Our approach can be extended to other novel land-use options other than bioenergy. Coupling spatially explicit economic simulation models with environmental and ESS assessment models, we can contribute to a comprehensive impact assessment of novel and hardly deployed land-use options in terms of their effects on multiple ESS.

## Supporting Information

S1 File Tables supporting Fig 3. Provisioning and regulating ESS values for the economic (scenarios 1–4) and the policy-driven scenarios (scenarios 5–6) compared to the baseline scenario.
(DOCX)

S2 File Tables supporting Table 3 and Fig 5. Tables listing the set of potential explanatory variables for Table 3 and Fig 5 and the entire regression results for Fig 5.
(DOCX)

S1 Text Full model description of economic simulation model. This document contains the full description of the economic simulation model following the ODD+D protocol [40].
(DOCX)

## References

[1] IEA. Energy Technology Perspectives 2010: Scenarios and Strategies to 2050. Paris: International Energy Agency (IEA), 2010.

[2] Chum H, Faaij A, Moreira J, Berndes G, Dhamija P, Dong H, et al Bioenergy. Cambridge, United Kingdom and New York, NY, USA: 2011.

[3] Tilman D, Socolow R, Foley JA, Hill J, Larson E, Lynd L, et al Beneficial Biofuels—The Food, Energy, and Environment Trilemma. Science. 2009;325(5938):270–1. 19608900 10.1126/science.1177970

[4] Fitzherbert EB, Struebig MJ, Morel A, Danielsen F, Brühl CA, Donald PF, et al How will oil palm expansion affect biodiversity? Trends in Ecology and Evolution. 2008;23(10):538–45. 10.1016/j.tree.2008.06.012 18775582

[5] Lambin EF, Meyfroidt P. Global land use change, economic globalization, and the looming land scarcity. P Natl Acad Sci USA. 2011;108(9):3465–72. 10.1073/pnas.1100480108. WOS:000287844400010. PMC3048112 21321211

[6] Milner S, Holland RA, Lovett A, Sunnenberg G, Hastings A, Smith P, et al Potential impacts on ecosystem services of land use transitions to second generation bioenergy crops in GB. GCB Bioenergy. 2015. 10.1111/gcbb.12263 PMC4974899 27547244

[7] Holland RA, Eigenbrod F, Muggeridge A, Brown G, Clarke D, Taylor G. A synthesis of the ecosystem services impact of second generation bioenergy crop production. Renewable and Sustainable Energy Reviews. 2015;46:30–40.

[8] Valentine J, Clifton-Brown J, Hastings A, Robson P, Allison G, Smith P. Food vs. fuel: the use of land for lignocellulosic next generation' energy crops that minimize competition with primary food production. Global Change Biology Bioenergy. 2012;4(1):1–19.

[9] Tilman D, Socolow R, Foley JA, Hill J, Larson E, Lynd L, et al Beneficial Biofuels-The Food, Energy, and Environment Trilemma. Science. 2009;325(5938):270–1. 19608900 10.1126/science.1177970

[10] Board MEA. Ecosystems and human well-being In: Hassan R, Scholes R, Ash N, editors. Ecosystems and human well-being: current state and trends: findings of the condition and trends working group: Island Press, Washington, DC; 2005.

[11] Power AG. Ecosystem services and agriculture: tradeoffs and synergies. Philos T R Soc B. 2010;365(1554):2959–71. 10.1098/rstb.2010.0143. WOS:000281922800015. PMC2935121 20713396

[12] Raudsepp-Hearne C, Peterson GD, Bennett EM. Ecosystem service bundles for analyzing tradeoffs in diverse landscapes. P Natl Acad Sci USA. 2010;107(11):5242–7. 10.1073/pnas.0907284107. WOS:000275714300077. PMC2841950 20194739

[13] Werling BP, Dickson TL, Isaacs R, Gaines H, Gratton C, Gross KL, et al Perennial grasslands enhance biodiversity and multiple ecosystem services in bioenergy landscapes. P Natl Acad Sci USA. 2014;111(4):1652–7. 10.1073/pnas.1309492111. WOS:000330231100092. PMC3910622 24474791

[14] Hastings A, Tallis MJ, Casella E, Matthews RW, Henshall PA, Milner S, et al The technical potential of Great Britain to produce ligno-cellulosic biomass for bioenergy in current and future climates. GCB Bioenergy. 2014;6(2):108–22. 10.1111/gcbb.12103

[15] Njakou Djomo S, Ac A, Zenone T, De Groote T, Bergante S, Facciotto G, et al Energy performances of intensive and extensive short rotation cropping systems for woody biomass production in the EU. Renewable and Sustainable Energy Reviews. 2015;41(0):845–54. 10.1016/j.rser.2014.08.058

[16] Sage R, Cunningham M, Boatman N. Birds in willow short-rotation coppice compared to other arable crops in central England and a review of bird census data from energy crops in the UK. Ibis. 2006;148:184–97.

[17] Rowe RL, Hanley ME, Goulson D, Clarke DJ, Doncaster CP, Taylor G. Potential benefits of commercial willow Short Rotation Coppice (SRC) for farm-scale plant and invertebrate communities in the agri-environment. Biomass Bioenerg. 2011;35(1):325–36.

[18] Makeschin F. Effects of Energy Forestry on Soils. Biomass Bioenerg. 1994;6(1–2):63–79. 10.1016/0961-9534(94)90086-8. WOS:A1994NU79000008.

[19] Schmidt-Walter P, Lamersdorf NP. Biomass Production with Willow and Poplar Short Rotation Coppices on Sensitive Areas-the Impact on Nitrate Leaching and Groundwater Recharge in a Drinking Water Catchment near Hanover, Germany. BioEnergy Research. 2012;5(3):546–62.

[20] Pe'er G, Dicks LV, Visconti P, Arlettaz R, Baldi A, Benton TG, et al Agriculture policy. EU agricultural reform fails on biodiversity. Science. 2014;344(6188):1090–2. 10.1126/science.1253425. WOS:000336791200023. 24904142

[21] Drossart I, Mühlenhoff J. Holzenergie Bedeutung, Potenziale, Herausforderungen Agentur für Erneuerbare Energien, 2013.

[22] Kraxner F, Nordström E-M, Havlík P, Gusti M, Mosnier A, Frank S, et al Global bioenergy scenarios—Future forest development, land-use implications, and trade-offs. Biomass and Bioenergy. 2013;57(0):86–96.

[23] Meehan TD, Gratton C, Diehl E, Hunt ND, Mooney DF, Ventura SJ, et al Ecosystem-Service Tradeoffs Associated with Switching from Annual to Perennial Energy Crops in Riparian Zones of the US Midwest. PLOS ONE. 2013;8(11):e80093 10.1371/journal.pone.0080093 24223215 PMC3819318

[24] Aust C, Schweier J, Brodbeck F, Sauter UH, Becker G, Schnitzler JP. Land availability and potential biomass production with poplar and willow short rotation coppices in Germany. GCB Bioenergy. 2014;6(5):521–33.

[25] Tölle MH, Gutjahr O, Busch G, Thiele JC. Increasing bioenergy production on arable land: Does the regional and local climate respond? Germany as a case study. Journal of Geophysical Research: Atmospheres. 2014;119(6):2711–24.

[26] Asbjornsen H, Hernandez-Santana V, Liebman M, Bayala J, Chen J, Helmers M, et al Targeting perennial vegetation in agricultural landscapes for enhancing ecosystem services. Renew Agr Food Syst. 2014;29(2):101–25.

[27] Parker DC, Hessl A, Davis SC. Complexity, land-use modeling, and the human dimension: Fundamental challenges for mapping unknown outcome spaces. Geoforum. 2008;39(2):789–804. 10.1016/j.geoforum.2007.05.005. WOS:000254925000026.

[28] Le QB, Seidl R, Scholz RW. Feedback loops and types of adaptation in the modelling of land-use decisions in an agent-based simulation. Environ Modell Softw. 2012;27–28:83–96. 10.1016/j.envsoft.2011.09.002. WOS:000298457000008.

[29] Lupp G, Steinhäußer R, Starick A, Gies M, Bastian O, Albrechtb J. Forcing Germany’s renewable energy targets by increased energy crop production: A challenge for regulation to secure sustainable landuse practices. Land Use Policy. 2014;36:296–306.

[30] Meyer MA, Priess JA. Indicators of bioenergy-related certification schemes—An analysis of the quality and comprehensiveness for assessing local/regional environmental impacts. Biomass and Bioenergy. 2014;65:151–69.

[31] Koellner T, Geyer R. Global land use impact assessment on biodiversity and ecosystem services in LCA. Int J Life Cycle Assess. 2013;18(6):1185–7. 10.1007/s11367-013-0580-6

[32] Jäckel G, Zink M, Marx A. Aufbereitung von gemessenen und simulierten Klimadaten; 2012.

[33] Altermann M, Ruske R. Die Region Leipzig-Halle-Bitterfeld: Geologic Lund Borden In: Feldmann R H K.; Auge H.; Flachowsky J.; Klotz S.; Krönert R., editor. Regeneration und nachhaltige Landnutzung—Konzepte für belastete Regionen: Springer, Berlin, Heidelberg; 1997.

[34] TU Dresden/AgroForNet. Kurzumtriebsplantagen in Sachsen. 2013. Available: http://www.energieholz-portal.de/257-0-KUP-in-Sachsen.html. Accessed November 04 2015.

[35] Das S, Eichhorn M, Hopffgarten MV, Lang E, Priess J, Thrän D. Spatial Analysis of the Potential of District Heating from Existing Bioenergy Installations in Germany; 2012; Milan ETA-Florence Renewable Energies.

[36] European Environment Agency (EEA). Corine Land Cover 2006 raster data; 2013. Accessed: http://www.eea.europa.eu/data-and-maps/data/corine-land-cover-2006-raster-3.

[37] Wochele S, Priess J, Thrän D, O’Keeffe S. Crop allocation model “CRAM”—an approach for dealing with biomass supply from arable land as part of a life cycle inventory. In: Hoffmann C, Baxter D., Maniatis K., Grassi A., Helm P., editor; 2014; Hamburg ETA-Florence Renewable Energies.

[38] Wochele-Marx S, Lang E, Pomm S, Das S, Priess J. Central Germany GIS dataset; 2015. Database: figshare. Accessed: https://figshare.com/articles/Central_Germany_GIS_dataset/1318765/2.

[39] Weise H. Land use change in the context of bioenergy production: impact assessment using agent-based modelling [PhD Thesis]: University of Osnabrück; 2014.

[40] Müller B, Bohn F, Dreßler G, Groeneveld J, Klassert C, Martin R, et al Describing human decisions in agent-based models—ODD + D, an extension of the ODD protocol. Environmental Modelling & Software. 2013;48:37–48.

[41] Grimm V, Berger U, Bastiansen F, Eliassen S, Ginot V, Giske J, et al A standard protocol for describing individual-based and agent-based models. Ecological Modelling. 2006;198(1–2):115–26.

[42] Grimm V, Berger U, DeAngelis DL, Polhill JG, Giske J, Railsback SF. The ODD protocol: A review and first update. Ecological Modelling. 2010;221(23):2760–8.

[43] LfULG. Auswertekarten Bodenschutz 1:50.000; 2012. Accessed: http://www.umwelt.sachsen.de/umwelt/boden/26192.htm.

[44] Renting H, Rossing WAH, Groot JCJ, Van der Ploeg JD, Laurent C, Perraud D, et al Exploring multifunctional agriculture. A review of conceptual approaches and prospects for an integrative transitional framework. J Environ Manage. 2009;90:S112–S23. 10.1016/j.jenvman.2008.11.014. WOS:000266651100002. 19121889

[45] Brown C, Bakam I, Smith P, Matthews R. An agent-based modelling approach to evaluate factors influencing bioenergy crop adoption in North East Scotland. GCB Bioenergy. 2015.

[46] Skodawessely, Pretzsch, Bemmann. Beratungshandbuch zu KUP: Eigenverlag der TU Dresden; 2010.

[47] AGROWOOD. 2015. Available: http://www.agrowood.de/ergebnisse.php. Accessed November 04 2015.

[48] Engelkamp P, Sell F. Einführung in die Volkswirtschaftslehre: Springer, Berlin, Heidelberg; 2007.

[49] Mankiw NG, Taylor MP. Economics: Thomson Learning Services, Toronto; 2006.

[50] Lawler JJ, Lewis DJ, Nelson E, Plantinga AJ, Polasky S, Withey JC, et al Projected land-use change impacts on ecosystem services in the United States. P Natl Acad Sci USA. 2014;111(20):7492–7. 10.1073/pnas.1405557111. WOS:000336168100070. PMC4034212 24799685

[51] FNR. Basisdaten Bioenergie Deutschland. Gülzow: Fachagentur Nachwachsende Rohstoffe e. V., 2013.

[52] Matzenberger J, Kranzl L, Tromborg E, Junginger M, Daioglou V, Sheng Goh C, et al Future perspectives of international bioenergy trade. Renewable and Sustainable Energy Reviews. 2015;43(0):926–41. 10.1016/j.rser.2014.10.106

[53] Becker G, Brunsmeier M. Konkurrenz zwischen stofflicher und energetischer Holznutzung—auch eine Frage der Allokation. Schweizerische Zeitschrift fur Forstwesen. 2013;164(12):382–8.

[54] Edel M, Thraen D. The Economic Viability of Wood Energy Conversion Technologies in Germany. International Journal of Forest Engineering. 2012;23(2):102–13.

[55] StaLa Sachsen. Bodennutzung und Ernte im Freistaat Sachsen—Feldfrüchte, Obst, Wein und Gemüse 2006. Kamenz: Statistisches Landesamt des Freistaates Sachsen, 2007.

[56] TLS. Ernte- und Betriebsberichterstattung—Feldfrüchte und Grünland in Thüringen—2006. Erfurt: Thüringer Landesamt für Statistik, 2007.

[57] BGR. Ackerbauliches Ertragspotential der Böden in Deutschland 1: 1 000 000; 2014. Accessed: http://www.bgr.bund.de/DE/Themen/Boden/Ressourcenbewertung-management/Ertragspotential/Ertragspotential_node.html.

[58] Ali W. Modelling of Biomass Production Potential of Poplar in Short Rotation Plantations on Agricultural Lands of Saxony, Germany [PhD Thesis]: Dresden University of Technology; 2009.

[59] Eggleston HS, Buendia L, Miwa K, Ngara T, Tanabe K. Guidelines for National Greenhouse Gas Inventories National Greenhouse Gas Inventories Programme. Institute for Global Environmental Strategies, 2006.

[60] Nelson E, Mendoza G, Regetz J, Polasky S, Tallis H, Cameron D, et al Modeling multiple ecosystem services, biodiversity conservation, commodity production, and tradeoffs at landscape scales. Frontiers in Ecology and the Environment. 2009;7(1):4–11. 10.1890/080023

[61] Kareiva P, Tallis H, Ricketts TH, Daily GC, Polasky S. Natural Capital: Theory and Practice of Mapping Ecosystem Services: Oxford Oxford University Press; 2011.

[62] Tallis HT, Ricketts T, Guerry AD, Wood SA, Sharp R, Nelson E, et al. InVEST 2.5.6 User’s Guide. Stanford: 2013.

[63] Wischmeier WH, Smith DD. Predicting rainfall erosion losses: A guide to conservation planning. Washington, DC: 1978.

[64] Meyer MA, Seppelt R, Witing F, Priess JA. Making environmental assessments of biomass production systems comparable worldwide. Environmental Research Letters. 2016;11(3):034005.

[65] Alkemade R, van Oorschot M, Miles L, Nellemann C, Bakkenes M, ten Brink B. GLOBIO3: A Framework to Investigate Options for Reducing Global Terrestrial Biodiversity Loss. Ecosystems. 2009;12(3):374–90. 10.1007/s10021-009-9229-5

[66] Mouchet MA, Lamarque P, Martin-Lopez B, Crouzat E, Gos P, Byczek C, et al An interdisciplinary methodological guide for quantifying associations between ecosystem services. Global Environ Chang. 2014;28:298–308. 10.1016/j.gloenvcha.2014.07.012. WOS:000343839100026.

[67] R Development Core Team. R: A language and environment for statistical computing. 2009 Available: http://www.r-project.org/. Accessed November 04 2015.

[68] Manning P, Taylor G, Hanley EM. Bioenergy, Food Production and Biodiversity—An Unlikely Alliance? GCB Bioenergy. 2015;7(4).

[69] Fürst C, Frank S, Witt A, Koschke L, Makeschin F. Assessment of the effects of forest land use strategies on the provision of ecosystem services at regional scale. J Environ Manage. 2013;127:S96–S116. 10.1016/j.jenvman.2012.09.020. WOS:000324227900011. 23158524

[70] Hellmann F, Verburg PH. Spatially explicit modelling of biofuel crops in Europe. Biomass and Bioenergy. 2011;35(6):2411–24.

[71] Kocoloski M, Griffin WM, Matthews HS. Impacts of facility size and location decisions on ethanol production cost. Energy Policy. 2011;39(1):47–56. 10.1016/j.enpol.2010.09.003. WOS:000285492000005.

[72] Vanloocke A, Bernacchi CJ, Twine TE. The impacts of Miscanthus x giganteus production on the Midwest US hydrologic cycle. Global Change Biology Bioenergy. 2010;2(4):180–91.

[73] Meyer MA, Chand T, Priess JA. Comparing bioenergy production sites in the Southeastern US regarding ecosystem service supply and demand. PLOS ONE. 2015;10(3):e0116336 10.1371/journal.pone.0116336 25768660 PMC4359142

[74] Holland JH. Complex adaptive systems. Daedalus—Journal of the American Academy of Arts and Science. 1992;121:17–30.

[75] Blumöhr T, Brandl M, Breitenfeld J, Dahl S, Führer J, Gabka D, et al Agrarstrukturen in Deutschland—Einheit in Vielfalt—Regionale Ergebnisse der Landwirtschaftszählung. Stuttgart: Statistisches Landesamt Baden-Württemberg, 2011.

[76] Glithero NJ, Wilson P, Ramsden SJ. Prospects for arable farm uptake of Short Rotation Coppice willow and miscanthus in England. Applied Energy. 2013;107(0):209–18. 23825896 10.1016/j.apenergy.2013.02.032 PMC3688319

[77] Sattler C, Nagel UJ. Factors affecting farmers’ acceptance of conservation measures—A case study from north-eastern Germany. Land Use Policy. 2010;27:70–7.

[78] Sherrington C, Bartley J, Moran D. Farm-level constraints on the domestic supply of perennial energy crops in the UK. Energy Policy. 2008;36(7):2504–12.

[79] Berger T. Agent-based spatial models applied to agriculture: a simulation tool for technology diffusion, resource use changes and policy analysis. Agr Econ-Blackwell. 2001;25(2–3):245–60. 10.1111/j.1574-0862.2001.tb00205.x. WOS:000172267200012.

[80] Roßberg D, Michel V, Graf R, Neukampf R. Definition von Boden-Klima-Raumen fur die Bundesrepublik Deutschland. Nachrichtenblatt des deutschen Pflanzenschutzdienstes. 2007;59(7):155–61.

[81] Meyfroidt P, Lambin EF, Erb K-H, Hertel TW. Globalization of land use: distant drivers of land change and geographic displacement of land use. Curr Opin Env Sust. 2013;5(5):438–44. 10.1016/j.cosust.2013.04.003

[82] Amer M, Daim TU, Jetter A. A review of scenario planning. Futures. 2013;46:23–40. 10.1016/j.futures.2012.10.003. WOS:000316034700003.

[83] Lehner B, Verdin K, Jarvis A. New Global Hydrography Derived From Spaceborne Elevation Data. Eos, Transactions American Geophysical Union. 2008;89(10):93–4. 10.1029/2008eo100001

[84] LfULG. Potentielle natürliche Vegetation (pnV) in Sachsen; 2011. Accessed: http://www.umwelt.sachsen.de/umwelt/natur/24728.htm.

[85] FAO Geonetwork. Global map of monthly reference evapotranspiration—10 arc minutes (GeoLayer). 2014. Available: http://www.fao.org/geonetwork/srv/en/main.home. Accessed November 04 2015.

[86] Panagos P, Van Liedekerke M, Montarella L. The European Soil Database distribution version 2.0; 2006. Accessed: http://eusoils.jrc.ec.europa.eu/esdb_archive/ESDB/Index.htm.

[87] Bräunig A. Dokumentation zur Berechnung und Ableitung R-Faktor Sachsen; 2013.

[88] Bischoff R. K-Faktor (MMK 100); 2014. Accessed: http://www.umwelt.sachsen.de/umwelt/boden/27787.htm.

[89] Reckhow KH, Beaulac M, Simpson J. Modeling Phosphorous Loading and Lake Response under Uncertainty: A Manual and Compilation of Export Coefficients Washington, DC: 1980.

[90] SMUL. Erosionsminderung. 2013. Available: http://www.umwelt.sachsen.de/umwelt/4566.asp. Accessed November 04 2015.

[91] Fraver S, Wagner RG, Day M. Dynamics of coarse woody debris following gap harvesting in the Acadian forest of central Maine, U.S.A. Canadian Journal of Forest Research. 2002;32(12):2094–105. 10.1139/x02-131

[92] Bohn U, Hettwer C, Gollub G. Anwendung und Auswertung der Karte der natürlichen Vegetation Europas: Beiträge und Ergebnisse des internationalen Workshops auf der Insel Vilm: Bonn Deutschland / Bundesamt für Naturschutz; 2005. 452 p.

[93] Müller-Using S, Bartsch N. Decay dynamic of coarse and fine woody debris of a beech (Fagus sylvatica L.) forest in Central Germany. Eur J Forest Res. 2009;128(3):287–96.

[94] Wördehoff R, Spellmann H, Evers J, Nagel J. Kohlenstoffstudie Forst und Holz Niedersachsen: Göttingen Universitätsverlag Göttingen; 2011.

[95] Polley H, Henning P. Die Bundeswaldinventur in der Geodateninfrastruktur des Thünen-Instituts (BWI 2002). 2012. Available: https://gdi.ti.bund.de/geoserver/bwi_2009dt/ows?SERVICE=WFS&REQUEST=GetCapabilities. Accessed November 04 2015.

[96] Strogies M, Gniffke P. Berichterstattung unter der Klimarahmenkonvention der Vereinten Nationen und dem Kyoto-Protokoll 2012—Nationaler Inventarbericht zum Deutschen Treibhausgasinventar 1990–2010. Dessau: Umweltbundesamt, 2012.

[97] BGR. Nutzungsdifferenzierte Bodenübersichtskarte der Bundesrepublik Deutschland 1:1.000.000 (BÜK1000N) (Serie); 2013. Accessed: http://www.bgr.de/app/Produktblatt/show.php?productid=DE-PR-0989#group8.

[98] Priess J. Population scenarios for Central Germany; 2016. Database: figshare. Accessed: https://figshare.com/articles/Population_scenarios_for_Central_Germany/3082183/1.

[99] Priess JA, Heinze M, Egli L, Pomm S, Lang E, Masurowski F, et al. Assessing some of the social and environmental consequences of changes in population and settlement pattern expected until 2050 for a Central European region. Environ Modell Softw. under review.

[100] BKG. Digitales Basis-Landschaftsmodell—Basis-DLM; 2014. Accessed: http://www.geodatenzentrum.de/docpdf/basis-dlm-aaa.pdf.

[101] Builtjes P, Hendriks E, Koenen M, Schaap M, Banzhaf S, Kerschbaumer A, et al. Abschlussbericht zum UFOPLAN-Vorhaben FKZ 3707 64 200: Erfassung, Prognose und Bewertung von Stoffeinträgen und ihren Wirkungen in Deutschland (Modelling of Air Pollutants and Ecosystem Impact—MAPESI). 2011.

[102] Olson DM, Dinerstein E, Wikramanayake ED, Burgess ND, Powell GVN, Underwood EC, et al Terrestrial Ecoregions of the World: A New Map of Life on Earth: A new global map of terrestrial ecoregions provides an innovative tool for conserving biodiversity. BioScience. 2001;51(11):933–8. 10.1641/0006-3568(2001)051[0933:TEOTWA]2.0.CO;2

