# Supplementary material for: Assessing Regional-Scale Impacts of Short Rotation Coppices on Ecosystem Services by Modeling Land-Use Decisions
Source: PLoS One. 2016 Apr 15;11(4):e0153862. doi: 10.1371/journal.pone.0153862 (PMC4833342; doi:10.1371/journal.pone.0153862)
Supplement: S1 File — Provisioning and regulating ESS values for the economic (scenarios 1–4) and the policy-driven scenarios (scenarios 5–6) compared to the baseline scenario. (DOCX) [file pone.0153862.s001.docx]

# S1 File: Tables supporting Figure 3

Table A. Provisioning ESS values for the economic (scenarios 1-4) and the policy-driven scenarios (scenarios 5-6) are indicated compared to the baseline scenario (italics); the upper part shows the scenario regional price scenario and the lower part the local price scenario.

Table B. Regulating ESS values for the economic (scenarios 1-4) and the policy-driven scenarios (scenarios 5-6) are indicated compared to the baseline scenario (italics); the upper part shows the scenario regional price scenario and the lower part the local price scenario.
