# Supplementary material for: Assessing Regional-Scale Impacts of Short Rotation Coppices on Ecosystem Services by Modeling Land-Use Decisions
Source: PLoS One. 2016 Apr 15;11(4):e0153862. doi: 10.1371/journal.pone.0153862 (PMC4833342; doi:10.1371/journal.pone.0153862)
Supplement: S2 File — Tables listing the set of potential explanatory variables for Table 3 and Fig 5 and the entire regression results for Fig 5. (DOCX) [file pone.0153862.s002.docx]

# S2 File: Tables listing the set of potential explanatory variables for Table 3 and Fig. 5 and the entire regression results for Fig. 5

Table A. Potential variables to explain ESS cluster differences

| **Independent variable** | **Unit** | | **Methodological reference (data source)** |
| --- | --- | --- | --- |
| *Landscape composition* |  | | [Jones *et al.* [1](#_ENREF_1)] |
| Share of LU/LC classes in the neighborhood: |  | | [European Environment Agency (EEA) [2](#_ENREF_2)], [Wochele *et al.* [3](#_ENREF_3)], [Wochele-Marx *et al.* [4](#_ENREF_4)] |
| Forest | [%] | |  |
| Pasture | [%] | |  |
| SRC | [%] | |  |
| Cropland | [%] | |  |
| Urban | [%] | |  |
| Water | [%] | |  |
| Distance to stream | [m] | | [Kissel *et al.* [5](#_ENREF_5)] |
| *Naturalness* |  | |  |
| Urbanity | [*score*] | | [European Environment Agency (EEA) [2](#_ENREF_2)], [Wochele *et al.* [3](#_ENREF_3)], [Wochele-Marx *et al.* [4](#_ENREF_4)], [Meyer *et al.* [6](#_ENREF_6)] |
| *Topography* |  | | [Wrbka *et al.* [7](#_ENREF_7)] |
| Elevation | [m] | | [Lehner *et al.* [8](#_ENREF_8)] |
| Slope | [*%*] | | [Lehner *et al.* [8](#_ENREF_8)] |
| Curvature | [*score*] | | [Lehner *et al.* [8](#_ENREF_8)]) |
| Aspect | [°] | | [Lehner *et al.* [8](#_ENREF_8)] |
|  |  | |  |
| *Soil parameters* |  | | [Qiu and Turner [9](#_ENREF_9)] |
| Effective rooting depth | [mm] | | [Panagos *et al.* [10](#_ENREF_10)], [LfULG [11](#_ENREF_11)] |
| Available water holding capacity | [cm cm^-1^] | | [Panagos *et al.* [10](#_ENREF_10)], [LfULG [11](#_ENREF_11)] |
| Soil quality index (“Ackerzahl”) | [*score*] | | [LfULG [11](#_ENREF_11)] |
| Erodibility (K) | [t ha h ha^-1^ MJ^-1^ mm^-1^] | | [LfULG [11](#_ENREF_11)], [Bischoff [12](#_ENREF_12)] |
| *Climate* | |  |  |
| Precipitation | | [mm a^-1^] | [Jäckel *et al.* [13](#_ENREF_13)] |
| Reference Evapotranspiration (10 arc-min) | | [mm a^-1^] | [FAO Geonetwork [14](#_ENREF_14)] |
| Erosivity (R) | | [MJ mm ha^-1^ h^-1^ a^-1^] | [Bräunig [15](#_ENREF_15)] |

Table B. Factors characterizing ESS cluster 1 and 2 for scenario 4 (backward logistic regression). A positive value for the standardized β indicates that an explanatory variable is contributing to cluster 1; a negative value for the standardized β indicates that an explanatory variable is contributing to cluster 2. The likelihood ratio test showed a significant difference when the final model was compared to a null model (χ2= 3262.2, df = 15, p<2.2e-16). Comparing the final model with a model including x- and y-coordinates, the likelihood ratio test showed only a small difference (χ2= 85.498, df = 3, p<2.2e-16).

| **Explanatory variable** | **Stand. β** | **SE** | **z value** | **Pr(>\|z\|)** |  |
| --- | --- | --- | --- | --- | --- |
| (Intercept) | 5.1502 | 0.5214 | 9.877 | < 0.0001 | *** |
| ***Topography and soil parameters*** |  |  |  |  |  |
| Elevation [m] | -4.77 | 0.6076 | -7.85 | <0.0001 | *** |
| Slope [%] | 3.014 | 0.4638 | 6.498 | <0.0001 | *** |
| Aspect [*°*] | 0.2484 | 0.1725 | 1.44 | 0.149952 |  |
| Curvature [*score*] | -2.9456 | 0.5438 | -5.417 | <0.0001 | *** |
| Effective rooting depth [mm] | -3.2246 | 0.5502 | -5.86 | <0.0001 | *** |
| Erosivity (R) [MJ mm ha^-1^ h^-1^ a^-1^] | 1.5463 | 0.5905 | 2.619 | 0.008829 | ** |
| Available water holding capacity [cm cm^-1^] | -1.2713 | 0.8935 | -1.423 | 0.154792 |  |
| Soil quality index | 5.552 | 0.5954 | 9.324 | < 0.0001 | *** |
| ***Climate*** |  |  |  |  |  |
| Precipitation [mm] | 1.4046 | 0.3846 | 3.652 | 0.00026 | *** |
| Reference Evapotranspiration [mm a^-1^] | -0.6073 | 0.3212 | -1.891 | 0.05868 | . |
| ***Landscape composition*** |  |  |  |  |  |
| Forest, 5 km buffer [*%*] | -3.771 | 0.4805 | -7.848 | <0.0001 | *** |
| Pasture, 5 km buffer [*%*] | 1.1935 | 0.3157 | 3.781 | 0.000156 | *** |
| SRC, 5 km buffer [%] | -7.7616 | 0.34 | -22.83 | < 0.0001 | *** |
| Water, 5 km buffer [*%*] | -0.5853 | 0.3163 | -1.851 | 0.064214 | . |
| Distance to stream [m] | -1.0951 | 0.2177 | -5.03 | <0.0001 | *** |

Table C. Factors characterizing ESS cluster 1 and 3 for scenario 4 (backward logistic regression). A positive value for the standardized β indicates that an explanatory variable is contributing to cluster 1; a negative value for the standardized β indicates that an explanatory variable is contributing to cluster 3. The likelihood ratio test showed a significant difference when the final model was compared to a null model (χ2= 2676.4, df = 15, p<2.2e-16). Comparing the final model with a model including x- and y-coordinates, the likelihood ratio test showed only a small difference (χ2= 85.508, df = 3, p<2.2e-16).

| **Explanatory variable** | **Stand. β** | **SE** | **z value** | **Pr(>\|z\|)** |  |
| --- | --- | --- | --- | --- | --- |
| (Intercept) | 12.2106 | 0.8513 | 14.343 | < 0.0001 | *** |
| ***Topography and soil parameters*** |  |  |  |  |  |
| Elevation [m] | -4.5616 | 0.5515 | -8.271 | < 0.0001 | *** |
| Slope [%] | -4.806 | 0.5114 | -9.398 | < 0.0001 | *** |
| Curvature [*score*] | -4.5616 | 0.5515 | -8.271 | < 0.0001 | *** |
| Effective rooting depth [mm] | -7.3096 | 0.6962 | -10.499 | < 0.0001 | *** |
| Erosivity (R) [MJ mm ha^-1^ h^-1^ a^-1^] | -7.3068 | 0.7143 | -10.23 | < 0.0001 | *** |
| Erodibility (K) [t ha h ha^-1^ MJ^-1^ mm^-1^] | -2.5418 | 0.5072 | -5.011 | <0.0001 | *** |
| Available water holding capacity [cm cm^-1^] | -4.7678 | 1.2038 | -3.961 | <0.0001 | *** |
| Soil quality index | 9.3495 | 0.7668 | 12.194 | < 0.0001 | *** |
| ***Climate*** |  |  |  |  |  |
| Precipitation [mm] | 2.9361 | 0.4461 | 6.582 | <0.0001 | *** |
| Reference Evapotranspiration [mm a^-1^] | -3.4618 | 0.4631 | -7.476 | <0.0001 | *** |
| ***Landscape composition*** |  |  |  |  |  |
| Forest, 5 km buffer [*%*] | -6.4389 | 0.6306 | -10.21 | < 0.0001 | *** |
| Pasture, 5 km buffer [*%*] | 3.2612 | 0.4199 | 7.767 | <0.0001 | *** |
| SRC, 5 km buffer [*%*] | -2.5557 | 0.3863 | -6.616 | <0.0001 | *** |
| Urban, 5 km buffer [*%*] | 1.2493 | 0.6562 | 1.904 | 0.05694 | . |
| Water, 5 km buffer [*%*] | -0.8468 | 0.3011 | -2.812 | 0.00492 | ** |
| Distance to stream [m] | -1.653 | 0.2814 | -5.873 | <0.0001 | *** |

Table D. Factors characterizing ESS cluster 1 and 4 for scenario 4 (backward logistic regression). A positive value for the standardized β indicates that an explanatory variable is contributing to cluster 1; a negative value for the standardized β indicates that an explanatory variable is contributing to cluster 4. The likelihood ratio test showed a significant difference when the final model was compared to a null model (χ2= 1885.4, df = 11, p<2.2e-16). Comparing the final model with a model including x- and y-coordinates, the likelihood ratio test showed only a small difference (χ2= 13.691, df = 3, p=0.003357).

| **Explanatory variable** | **Stand. β** | **SE** | **z value** | **Pr(>\|z\|)** |  |
| --- | --- | --- | --- | --- | --- |
| (Intercept) | 1.6243 | 0.3484 | 4.662 | <0.0001 | *** |
| ***Topography and soil parameters*** |  |  |  |  |  |
| Slope [%] | 1.2629 | 0.3618 | 3.491 | 0.000482 | *** |
| Aspect [*°*] | -0.3112 | 0.1484 | -2.097 | 0.036033 | * |
| Curvature [*score*] | -1.2291 | 0.4226 | -2.909 | 0.003631 | ** |
| Effective rooting depth [mm] | -3.813 | 0.4469 | -8.532 | < 0.0001 | *** |
| Erodibility (K) [t ha h ha^-1^ MJ^-1^ mm^-1^] | 1.0086 | 0.28 | 3.602 | 0.000316 | *** |
| Soil quality index | 4.6556 | 0.4595 | 10.132 | < 0.0001 | *** |
| ***Climate*** |  |  |  |  |  |
| Precipitation [mm] | 0.7629 | 0.2363 | 3.229 | 0.001241 | ** |
| ***Landscape composition*** |  |  |  |  |  |
| Forest, 5 km buffer [*%*] | -1.9671 | 0.2927 | -6.721 | <0.0001 | *** |
| SRC, 5 km buffer [*%*] | -4.111 | 0.2258 | -18.206 | < 0.0001 | *** |
| Water, 5 km buffer [*%*] | 0.7133 | 0.2316 | 3.079 | 0.002076 | ** |
| Distance to stream [m] | -0.4168 | 0.1734 | -2.404 | 0.016197 | * |

Table E. Factors characterizing ESS cluster 1 and 5 for scenario 4 (backward logistic regression). A positive value for the standardized β indicates that an explanatory variable is contributing to cluster 1; a negative value for the standardized β indicates that an explanatory variable is contributing to cluster 5. The likelihood ratio test showed a significant difference when the final model was compared to a null model (χ2= 1445.4, df = 13, p<2.2e-16).

| **Explanatory variable** | **Stand. β** | **SE** | **z value** | **Pr(>\|z\|)** |  |
| --- | --- | --- | --- | --- | --- |
| (Intercept) | -6.583 | 0.6293 | -10.461 | < 0.0001 | *** |
| ***Topography and soil parameters*** |  |  |  |  |  |
| Elevation [m] | 5.3039 | 0.6956 | 7.625 | <0.0001 | *** |
| Slope [%] | 2.9376 | 0.5522 | 5.32 | <0.0001 | *** |
| Aspect [*°*] | -0.6175 | 0.2155 | -2.865 | 0.004168 | ** |
| Curvature [*score*] | 6.1831 | 0.6694 | 9.236 | < 0.0001 | *** |
| Effective rooting depth [mm] | 2.2987 | 0.3414 | 6.734 | <0.0001 | *** |
| Available water holding capacity [cm cm^-1^] | 12.0156 | 0.9828 | 12.226 | < 0.0001 | *** |
| ***Climate*** |  |  |  |  |  |
| Precipitation [mm] | -2.5332 | 0.4769 | -5.312 | <0.0001 | *** |
| Reference Evapotranspiration [mm a^-1^] | 1.3533 | 0.3957 | 3.42 | 0.000627 | *** |
| ***Landscape composition*** |  |  |  |  |  |
| Forest, 5 km buffer [*%*] | -0.6897 | 0.4264 | -1.617 | 0.105782 |  |
| SRC, 5 km buffer [*%*] | -0.6687 | 0.2887 | -2.316 | 0.020562 | * |
| Urban, 5 km buffer [*%*] | -2.9291 | 0.31 | -9.447 | < 0.0001 | *** |
| Water, 5 km buffer [*%*] | 1.9425 | 0.3846 | 5.051 | <0.0001 | *** |
| Distance to stream [m] | -0.683 | 0.2542 | -2.687 | 0.007202 | ** |

Table F. Factors characterizing ESS cluster 1 and 6 for scenario 4 (backward logistic regression). A positive value for the standardized β indicates that an explanatory variable is contributing to cluster 1; a negative value for the standardized β indicates that an explanatory variable is contributing to cluster 6. The likelihood ratio test showed a significant difference when the final model was compared to a null model (χ2= 1600.2, df = 12, p<2.2e-16). Comparing the final model with a model including x- and y-coordinates, the likelihood ratio test showed only a small difference (χ2= 37.034, df = 3, p= 4.526e-08).

| **Explanatory variable** | **Stand. β** | **SE** | **z value** | **Pr(>\|z\|)** |  |
| --- | --- | --- | --- | --- | --- |
| (Intercept) | 8.8382 | 0.583 | 15.159 | < 0.0001 | *** |
| ***Topography and soil parameters*** |  |  |  |  |  |
| Elevation [m] | -3.9391 | 0.6124 | -6.432 | <0.0001 | *** |
| Slope [%] | -8.6778 | 0.4189 | -20.713 | < 0.0001 | *** |
| Curvature [*score*] | -0.7426 | 0.4466 | -1.663 | 0.096344 | . |
| Erodibility (K) [t ha h ha^-1^ MJ^-1^ mm^-1^] | 1.0074 | 0.3419 | 2.946 | 0.003215 | ** |
| Erosivity (R) [MJ mm ha^-1^ h^-1^ a^-1^] | -1.6357 | 0.679 | -2.409 | 0.016004 | * |
| Available water holding capacity [cm cm^-1^] | -2.9583 | 0.7628 | -3.878 | 0.000105 | *** |
| ***Climate*** |  |  |  |  |  |
| Precipitation [mm] | 2.4498 | 0.3919 | 6.251 | <0.0001 | *** |
| Reference Evapotranspiration [mm a^-1^] | -4.4756 | 0.3909 | -11.451 | < 0.0001 | *** |
| ***Landscape composition*** |  |  |  |  |  |
| Forest, 5 km buffer [*%*] | -3.9054 | 0.4851 | -8.05 | <0.0001 | *** |
| Pasture, 5 km buffer [*%*] | 1.5455 | 0.3502 | 4.414 | <0.0001 | *** |
| SRC, 5 km buffer [*%*] | -2.1195 | 0.2992 | -7.084 | <0.0001 | *** |
| Urban, 5 km buffer [*%*] | -2.4177 | 0.3991 | -6.058 | <0.0001 | *** |

Table G. Factors characterizing ESS cluster 2 and 3 for scenario 4 (backward logistic regression). A positive value for the standardized β indicates that an explanatory variable is contributing to cluster 2; a negative value for the standardized β indicates that an explanatory variable is contributing to cluster 3. The likelihood ratio test showed a significant difference when the final model was compared to a null model (χ2= 1466, df = 14, p<2.2e-16). Comparing the final model with a model including x- and y-coordinates, the likelihood ratio test showed only a small difference (χ2= 133.95, df = 3, p<2.2e-16).

| **Explanatory variable** | **Stand. β** | **SE** | **z value** | **Pr(>\|z\|)** |  |
| --- | --- | --- | --- | --- | --- |
| (Intercept) | 4.7764 | 0.7592 | 6.291 | <0.0001 | *** |
| ***Topography and soil parameters*** |  |  |  |  |  |
| Elevation [m] | -2.9234 | 0.72 | -4.061 | <0.0001 | *** |
| Slope [%] | -6.819 | 0.5473 | -12.46 | < 0.0001 | *** |
| Curvature [*score*] | -1.3193 | 0.5751 | -2.294 | 0.021804 | * |
| Effective rooting depth [mm] | -1.4654 | 0.6322 | -2.318 | 0.020444 | * |
| Erodibility (K) [t ha h ha^-1^ MJ^-1^ mm^-1^] | -1.8041 | 0.497 | -3.63 | 0.000283 | *** |
| Erosivity (R) [MJ mm ha^-1^ h^-1^ a^-1^] | -5.9939 | 0.7658 | -7.827 | <0.0001 | *** |
| Available water holding capacity [cm cm^-1^] | -1.9397 | 0.8838 | -2.195 | 0.000985 | * |
| Soil quality index | 2.3024 | 0.6988 | 3.295 | <0.0001 | *** |
| ***Climate*** |  |  |  |  |  |
| Precipitation [mm] | 2.9945 | 0.436 | 6.869 | <0.0001 | *** |
| Reference Evapotranspiration [mm a^-1^] | -3.8305 | 0.5029 | -7.618 | <0.0001 | *** |
| ***Landscape composition*** |  |  |  |  |  |
| Pasture, 5 km buffer [*%*] | 3.467 | 0.3238 | 10.708 | < 0.0001 | *** |
| SRC, 5 km buffer [*%*] | 6.6071 | 0.4044 | 16.338 | < 0.0001 | *** |
| Water, 5 km buffer [*%*] | -0.6291 | 0.347 | -1.813 | 0.069865 | . |
| Urban, 5 km buffer [*%*] | 2.8488 | 0.7024 | 4.056 | <0.0001 | *** |

Table H. Factors characterizing ESS cluster 2 and 4 for scenario 4 (backward logistic regression). A positive value for the standardized β indicates that an explanatory variable is contributing to cluster 2; a negative value for the standardized β indicates that an explanatory variable is contributing to cluster 4. The likelihood ratio test showed a significant difference when the final model was compared to a null model (χ2= 609.39.8, df = 15, p<2.2e-16). Comparing the final model with a model including x- and y-coordinates, the likelihood ratio test showed only a small difference (χ2= 30.01, df = 3, p=1.373e-06).

| **Explanatory variable** | **Stand. β** | **SE** | **z value** | **Pr(>\|z\|)** |  |
| --- | --- | --- | --- | --- | --- |
| (Intercept) | -0.6899 | 0.5579 | -1.237 | 0.216182 |  |
| ***Topography and soil parameters*** |  |  |  |  |  |
| Elevation [m] | 4.0458 | 0.5075 | 7.973 | <0.0001 | *** |
| Slope [%] | -2.0923 | 0.4452 | -4.699 | <0.0001 | *** |
| Aspect [°] | -0.3247 | 0.1553 | -2.091 | 0.036508 | * |
| Curvature [*score*] | 2.2409 | 0.4753 | 4.715 | <0.0001 | *** |
| Erodibility (K) [t ha h ha^-1^ MJ^-1^ mm^-1^] | 0.9522 | 0.3431 | 2.776 | 0.005511 | ** |
| Erosivity (R) [MJ mm ha^-1^ h^-1^ a^-1^] | -1.7973 | 0.4962 | -3.622 | 0.000293 | *** |
| Soil quality index | -2.0799 | 0.3625 | -5.737 | <0.0001 | *** |
| ***Climate*** |  |  |  |  |  |
| Precipitation [mm] | -0.6322 | 0.3272 | -1.932 | 0.053347 | . |
| Reference Evapotranspiration [mm a^-1^] | 0.708 | 0.3247 | 2.181 | 0.029212 | * |
| ***Landscape composition*** |  |  |  |  |  |
| Pasture, 5 km buffer [*%*] | -2.2926 | 0.2347 | -9.77 | < 0.0001 | *** |
| SRC, 5 km buffer [*%*] | 1.794 | 0.3398 | 5.28 | <0.0001 | *** |
| Cropland, 5 km buffer [*%*] | -2.1915 | 0.447 | -4.902 | <0.0001 | *** |
| Water, 5 km buffer [*%*] | 0.9181 | 0.3119 | 2.944 | 0.003242 | ** |
| Urban, 5 km buffer [*%*] | -1.48 | 0.4783 | -3.094 | 0.001975 | ** |
| Distance to stream [m] | 0.7341 | 0.1846 | 3.976 | <0.0001 | *** |

Table I. Factors characterizing ESS cluster 2 and 5 for scenario 4 (backward logistic regression). A positive value for the standardized β indicates that an explanatory variable is contributing to cluster 2; a negative value for the standardized β indicates that an explanatory variable is contributing to cluster 5. The likelihood ratio test showed a significant difference when the final model was compared to a null model (χ2= 1912.3.7, df = 16, p<2.2e-16). Comparing the final model with a model including x- and y-coordinates, the likelihood ratio test showed only a small difference (χ2= 72.888, df = 3, p= 1.027e-15).

| **Explanatory variable** | **Stand. β** | **SE** | **z value** | **Pr(>\|z\|)** |  |
| --- | --- | --- | --- | --- | --- |
| (Intercept) | -13.8425 | 0.9449 | -14.65 | < 0.0001 | *** |
| ***Topography and soil parameters*** |  |  |  |  |  |
| Elevation [m] | 10.2349 | 0.9931 | 10.306 | < 0.0001 | *** |
| Slope [%] | 1.9034 | 0.7526 | 2.529 | 0.01144 | * |
| Aspect [°] | -0.8473 | 0.2715 | -3.12 | 0.00181 | ** |
| Curvature [*score*] | 10.2743 | 0.9209 | 11.157 | < 0.0001 | *** |
| Effective rooting depth [mm] | 6.6289 | 0.8505 | 7.794 | <0.0001 | *** |
| Erodibility (K) [t ha h ha^-1^ MJ^-1^ mm^-1^] | 2.9127 | 0.6411 | 4.543 | <0.0001 | *** |
| Erosivity (R) [MJ mm ha^-1^ h^-1^ a^-1^] | -2.5054 | 0.9158 | -2.736 | 0.00623 | ** |
| Available water holding capacity [cm cm^-1^] | 15.5389 | 2.4739 | 6.281 | <0.0001 | *** |
| Soil quality index | -7.1422 | 1.1338 | -6.299 | <0.0001 | *** |
| ***Climate*** |  |  |  |  |  |
| Precipitation [mm] | -4.1262 | 0.5983 | -6.897 | <0.0001 | *** |
| Reference Evapotranspiration [mm a^-1^] | 0.9847 | 0.5301 | 1.858 | 0.06323 | . |
| ***Landscape composition*** |  |  |  |  |  |
| Forest, 5 km buffer [*%*] | 3.2695 | 0.6201 | 5.272 | <0.0001 |  |
| SRC, 5 km buffer [*%*] | 7.0331 | 0.4659 | 15.095 | < 0.0001 | *** |
| Urban, 5 km buffer [*%*] | -3.566 | 0.6424 | -5.551 | <0.0001 | *** |
| Water, 5 km buffer [*%*] | 3.518 | 0.5055 | 6.959 | <0.0001 | *** |
| Distance to stream [m] | 1.3894 | 0.3118 | 4.455 | <0.0001 | *** |

Table J. Factors characterizing ESS cluster 2 and 6 for scenario 4 (backward logistic regression). A positive value for the standardized β indicates that an explanatory variable is contributing to cluster 2; a negative value for the standardized β indicates that an explanatory variable is contributing to cluster 6. The likelihood ratio test showed a significant difference when the final model was compared to a null model (χ2= 1710.2, df = 12, p<2.2e-16). Comparing the final model with a model including x- and y-coordinates, the likelihood ratio test showed only a small and insignificant difference (χ2= 5.522, df = 3, p= 0.1373).

| **Explanatory variable** | **Stand. β** | **SE** | **z value** | **Pr(>\|z\|)** |  |
| --- | --- | --- | --- | --- | --- |
| (Intercept) | 4.8578 | 0.6886 | 7.055 | <0.0001 | *** |
| ***Topography and soil parameters*** |  |  |  |  |  |
| Slope [%] | -11.2392 | 0.548 | -20.509 | < 0.0001 | *** |
| Erodibility (K) [t ha h ha^-1^ MJ^-1^ mm^-1^] | -1.7174 | 0.4896 | -3.508 | 0.000452 | *** |
| Erosivity (R) [MJ mm ha^-1^ h^-1^ a^-1^] | -2.218 | 0.6798 | -3.263 | 0.001103 | ** |
| Effective rooting depth [mm] | -1.7088 | 0.6805 | -2.511 | 0.012036 | * |
| Available water holding capacity [cm cm^-1^] | -6.1165 | 0.9342 | -6.547 | <0.0001 | *** |
| Soil quality index | 1.4359 | 0.6994 | 2.053 | 0.040077 | * |
| ***Climate*** |  |  |  |  |  |
| Precipitation [mm] | 1.127 | 0.3907 | 2.885 | 0.003918 | ** |
| Reference Evapotranspiration [mm a^-1^] | -2.4961 | 0.4376 | -5.704 | <0.0001 | *** |
| ***Landscape composition*** |  |  |  |  |  |
| Forest, 5 km buffer [*%*] | 0.9692 | 0.5344 | 1.813 | 0.069763 | . |
| SRC, 5 km buffer [*%*] | 6.0974 | 0.4484 | 13.599 | < 0.0001 | *** |
| Urban, 5 km buffer [*%*] | -3.9211 | 0.6047 | -6.485 | <0.0001 | *** |
| Distance to stream [m] | 1.0508 | 0.258 | 4.073 | <0.0001 | *** |

Table K. Factors characterizing ESS cluster 3 and 4 for scenario 4 (backward logistic regression). A positive value for the standardized β indicates that an explanatory variable is contributing to cluster 3; a negative value for the standardized β indicates that an explanatory variable is contributing to cluster 4. The likelihood ratio test showed a significant difference when the final model was compared to a null model (χ2= 1646.8, df = 16, p<2.2e-16). Comparing the final model with a model including x- and y-coordinates, the likelihood ratio test showed a difference (χ2= 236.28, df = 3, p<2.2e-16).

| **Explanatory variable** | **Stand. β** | **SE** | **z value** | **Pr(>\|z\|)** |  |
| --- | --- | --- | --- | --- | --- |
| (Intercept) | -9.1475 | 0.8358 | -10.945 | < 0.0001 | *** |
| ***Topography and soil parameters*** |  |  |  |  |  |
| Elevation [m] | 6.0154 | 0.7269 | 8.275 | < 0.0001 | *** |
| Slope [%] | 5.9011 | 0.5347 | 11.036 | < 0.0001 | *** |
| Curvature [*score*] | 2.5667 | 0.5402 | 4.751 | <0.0001 | *** |
| Effective rooting depth [mm] | 2.0808 | 0.6827 | 3.048 | 0.002305 | ** |
| Erodibility (K) [t ha h ha^-1^ MJ^-1^ mm^-1^] | 2.9274 | 0.5185 | 5.646 | <0.0001 | *** |
| Erosivity (R) [MJ mm ha^-1^ h^-1^ a^-1^] | 4.4358 | 0.8118 | 5.464 | <0.0001 | *** |
| Available water holding capacity [cm cm^-1^] | 4.6746 | 1.4258 | 3.278 | 0.001044 | ** |
| Soil quality index | -4.8472 | 0.7893 | -6.141 | <0.0001 | *** |
| ***Climate*** |  |  |  |  |  |
| Precipitation [mm] | -3.4453 | 0.4665 | -7.386 | <0.0001 | *** |
| Reference Evapotranspiration [mm a^-1^] | 4.4468 | 0.5105 | 8.711 | < 0.0001 | *** |
| ***Landscape composition*** |  |  |  |  |  |
| Forest, 5 km buffer [*%*] | 3.2055 | 0.6464 | 4.959 | <0.0001 | *** |
| Pasture, 5 km buffer [*%*] | -4.1189 | 0.4227 | -9.745 | < 0.0001 | *** |
| SRC, 5 km buffer [*%*] | -2.136 | 0.4234 | -5.044 | <0.0001 | *** |
| Urban, 5 km buffer [*%*] | -1.6754 | 0.6936 | -2.416 | 0.01571 | * |
| Water, 5 km buffer [*%*] | 1.7689 | 0.318 | 5.562 | <0.0001 | *** |
| Distance to stream [m] | 0.901 | 0.2621 | 3.438 | 0.000587 | *** |

Table L. Factors characterizing ESS cluster 3 and 5 for scenario 4 (backward logistic regression). A positive value for the standardized β indicates that an explanatory variable is contributing to cluster 3; a negative value for the standardized β indicates that an explanatory variable is contributing to cluster 5. The likelihood ratio test showed a significant difference when the final model was compared to a null model (χ2= 1761.4, df = 14, p<2.2e-16). Comparing the final model with a model including x- and y-coordinates, the likelihood ratio test showed a small difference (χ2= 55.3, df = 3, p= 5.926e-12).

| **Explanatory variable** | **Stand. β** | **SE** | **z value** | **Pr(>\|z\|)** |  |
| --- | --- | --- | --- | --- | --- |
| (Intercept) | -18.2214 | 1.2309 | -14.804 | < 0.0001 | *** |
| ***Topography and soil parameters*** |  |  |  |  |  |
| Slope [%] | 7.5795 | 0.7841 | 9.667 | < 0.0001 | *** |
| Curvature [*score*] | 9.8788 | 0.8757 | 11.281 | < 0.0001 | *** |
| Effective rooting depth [mm] | 3.1985 | 0.6233 | 5.132 | <0.0001 | *** |
| Erodibility (K) [t ha h ha^-1^ MJ^-1^ mm^-1^] | 3.4341 | 0.6891 | 4.983 | <0.0001 | *** |
| Erosivity (R) [MJ mm ha^-1^ h^-1^ a^-1^] | 9.7706 | 1.1365 | 8.597 | < 0.0001 | *** |
| Available water holding capacity [cm cm^-1^] | 4.7929 | 1.6248 | 2.95 | 0.00318 | ** |
| ***Climate*** |  |  |  |  |  |
| Precipitation [mm] | -4.0495 | 0.6254 | -6.475 | <0.0001 | *** |
| Reference Evapotranspiration [mm a^-1^] | 3.6037 | 0.6621 | 5.443 | <0.0001 | *** |
| ***Landscape composition*** |  |  |  |  |  |
| Forest, 5 km buffer [*%*] | 7.161 | 0.9269 | 7.726 | <0.0001 | *** |
| Pasture, 5 km buffer [*%*] | -0.9041 | 0.5818 | -1.554 | 0.12019 |  |
| SRC, 5 km buffer [*%*] | 2.5835 | 0.5658 | 4.566 | <0.0001 | *** |
| Urban, 5 km buffer [*%*] | -5.9464 | 0.8748 | -6.797 | <0.0001 | *** |
| Water, 5 km buffer [*%*] | 2.8066 | 0.47 | 5.972 | <0.0001 | *** |
| Distance to stream [m] | 1.0941 | 0.3834 | 2.853 | 0.00432 | ** |

Table M. Factors characterizing ESS cluster 3 and 6 for scenario 4 (backward logistic regression). A positive value for the standardized β indicates that an explanatory variable is contributing to cluster 3; a negative value for the standardized β indicates that an explanatory variable is contributing to cluster 6. The likelihood ratio test showed a significant difference when the final model was compared to a null model (χ2= 813.53, df = 14, p<2.2e-16). Comparing the final model with a model including x- and y-coordinates, the likelihood ratio test showed a difference (χ2= 160.09, df = 3, p<2.2e-16).

| **Explanatory variable** | **Stand. β** | **SE** | **z value** | **Pr(>\|z\|)** |  |
| --- | --- | --- | --- | --- | --- |
| (Intercept) | -1.5289 | 0.793 | -1.928 | 0.053863 | . |
| ***Topography and soil parameters*** |  |  |  |  |  |
| Elevation [m] | 3.0177 | 0.7241 | 4.167 | <0.0001 | *** |
| Slope [%] | -2.4489 | 0.4227 | -5.793 | <0.0001 | *** |
| Aspect [°] | 0.3646 | 0.2336 | 1.561 | 0.118502 |  |
| Curvature [*score*] | 0.9358 | 0.4337 | 2.158 | 0.030955 | * |
| Effective rooting depth [mm] | 1.1254 | 0.4434 | 2.538 | 0.011147 | * |
| Erosivity (R) [MJ mm ha^-1^ h^-1^ a^-1^] | 3.398 | 0.7797 | 4.358 | <0.0001 | *** |
| Available water holding capacity [cm cm^-1^] | -3.5013 | 0.6796 | -5.152 | <0.0001 | *** |
| ***Climate*** |  |  |  |  |  |
| Precipitation [mm] | -1.4466 | 0.4595 | -3.148 | 0.001641 | ** |
| Reference Evapotranspiration [mm a^-1^] | 0.8246 | 0.4345 | 1.898 | 0.057714 | . |
| ***Landscape composition*** |  |  |  |  |  |
| Pasture, 5 km buffer [*%*] | -3.5496 | 0.3673 | -9.665 | < 0.0001 | *** |
| Cropland, 5 km buffer [*%*] | -0.6871 | 0.4512 | -1.523 | 0.127829 |  |
| Urban, 5 km buffer [*%*] | -6.7525 | 0.7074 | -9.545 | < 0.0001 | *** |
| Water, 5 km buffer [*%*] | 1.2738 | 0.3021 | 4.217 | <0.0001 | *** |
| Distance to stream [m] | 0.9859 | 0.2821 | 3.495 | 0.000474 | *** |

Table N. Factors characterizing ESS cluster 4 and 5 for scenario 4 (backward logistic regression). A positive value for the standardized β indicates that an explanatory variable is contributing to cluster 4; a negative value for the standardized β indicates that an explanatory variable is contributing to cluster 5. The likelihood ratio test showed a significant difference when the final model was compared to a null model (χ2= 1203.3, df = 10, p<2.2e-16). Comparing the final model with a model including x- and y-coordinates, the likelihood ratio test showed a small difference (χ2= 36.924, df = 3, p= 4.776e-8).

| **Explanatory variable** | **Stand. β** | **SE** | **z value** | **Pr(>\|z\|)** |  |
| --- | --- | --- | --- | --- | --- |
| (Intercept) | -7.7021 | 0.5501 | -14 | < 0.0001 | *** |
| ***Topography and soil parameters*** |  |  |  |  |  |
| Elevation [m] | 4.3332 | 0.5428 | 7.983 | <0.0001 | *** |
| Slope [%] | 2.6263 | 0.5686 | 4.619 | <0.0001 | *** |
| Curvature [*score*] | 7.0915 | 0.6944 | 10.213 | < 0.0001 | *** |
| Effective rooting depth [mm] | 2.6274 | 0.3883 | 6.766 | <0.0001 | *** |
| Available water holding capacity [cm cm^-1^] | 8.9751 | 1.0756 | 8.344 | < 0.0001 | *** |
| ***Climate*** |  |  |  |  |  |
| Precipitation [mm] | -2.584 | 0.441 | -5.86 | <0.0001 | *** |
| ***Landscape composition*** |  |  |  |  |  |
| Forest, 5 km buffer [*%*] | 0.7941 | 0.4513 | 1.76 | 0.0785 | . |
| SRC, 5 km buffer [*%*] | 3.7033 | 0.3259 | 11.363 | < 0.0001 | *** |
| Urban, 5 km buffer [*%*] | -4.3034 | 0.4135 | -10.408 | < 0.0001 | *** |
| Water, 5 km buffer [*%*] | 1.7223 | 0.384 | 4.485 | <0.0001 | *** |

Table O. Factors characterizing ESS cluster 4 and 6 for scenario 4 (backward logistic regression). A positive value for the standardized β indicates that an explanatory variable is contributing to cluster 4; a negative value for the standardized β indicates that an explanatory variable is contributing to cluster 6. The likelihood ratio test showed a significant difference when the final model was compared to a null model (χ2= 1169.6, df = 13, p<2.2e-16). Comparing the final model with a model including x- and y-coordinates, the likelihood ratio test showed a small difference (χ2= 10.682, df = 3, p= 0.01358).

| **Explanatory variable** | Estimate | Std. Error | z value | Pr(>\|z\|) |  |
| --- | --- | --- | --- | --- | --- |
| (Intercept) | 6.5561 | 0.6355 | 10.317 | < 0.0001 | *** |
| ***Topography and soil parameters*** |  |  |  |  |  |
| Elevation [m] | -3.9147 | 0.5301 | -7.384 | <0.0001 | *** |
| Slope [%] | -9.3879 | 0.453 | -20.725 | < 0.0001 | *** |
| Effective rooting depth [mm] | -1.702 | 0.629 | -2.706 | 0.006808 | ** |
| Erodibility (K) [t ha h ha^-1^ MJ^-1^ mm^-1^] | -2.146 | 0.4473 | -4.798 | <0.0001 | *** |
| Available water holding capacity [cm cm^-1^] | -9.0053 | 1.3415 | -6.713 | <0.0001 | *** |
| Soil quality index | 4.0277 | 0.6951 | 5.794 | <0.0001 | *** |
| ***Climate*** |  |  |  |  |  |
| Precipitation [mm] | 1.322 | 0.3994 | 3.31 | 0.000934 | *** |
| Reference Evapotranspiration [mm a^-1^] | -3.0745 | 0.3917 | -7.85 | <0.0001 | *** |
| ***Landscape composition*** |  |  |  |  |  |
| Forest, 5 km buffer [*%*] | -1.4619 | 0.5193 | -2.815 | 0.004876 | ** |
| Pasture, 5 km buffer [*%*] | 1.5603 | 0.3778 | 4.13 | <0.0001 | *** |
| SRC, 5 km buffer [*%*] | 2.594 | 0.326 | 7.956 | <0.0001 | *** |
| Urban, 5 km buffer [*%*] | -3.1592 | 0.5041 | -6.267 | <0.0001 | *** |
| Water, 5 km buffer [*%*] | -0.7262 | 0.3089 | -2.351 | 0.018721 | * |

## References

1. Jones KB, Neale A, Nash M, Van Remortel R, Wickham J, Riitters K, et al. Predicting nutrient and sediment loadings to streams from landscape metrics: A multiple watershed study from the United States Mid-Atlantic Region. Landscape Ecology. 2001;16(4):301-12. doi: 10.1023/a:1011175013278.

2. European Environment Agency (EEA). Corine Land Cover 2006 raster data; 2013. Accessed: <http://www.eea.europa.eu/data-and-maps/data/corine-land-cover-2006-raster-3>.

3. Wochele S, Priess J, Thrän D, O’Keeffe S. Crop allocation model “CRAM” - an approach for dealing with biomass supply from arable land as part of a life cycle inventory. In: Hoffmann C, Baxter, D., Maniatis, K., Grassi, A., Helm, P., editor; 2014; Hamburg. ETA-Florence Renewable Energies.

4. Wochele-Marx S, Lang E, Pomm S, Das S, Priess J. Central Germany GIS dataset; 2015. Database: figshare. Accessed: https://figshare.com/articles/Central_Germany_GIS_dataset/1318765/2.

5. Kissel M, Fuentes A, Deane-Drumnond C. The evolution of the human niche: assessing and describing the development of complex decision-making in the Pleistocene through an open-access, comparative database. Am J Phys Anthropol. 2015;156:188. PubMed PMID: WOS:000350594901249.

6. Meyer MA, Chand T, Priess JA. Comparing bioenergy production sites in the Southeastern US regarding ecosystem service supply and demand. PLOS ONE. 2015;10(3):e0116336.

7. Wrbka T, Erb KH, Schulz NB, Peterseil J, Hahn C, Haberl H. Linking pattern and process in cultural landscapes. An empirical study based on spatially explicit indicators. Land Use Policy. 2004;21(3):289-306. doi: 10.1016/j.landusepol.2003.10.012. PubMed PMID: WOS:000222834400009.

8. Lehner B, Verdin K, Jarvis A. New Global Hydrography Derived From Spaceborne Elevation Data. Eos, Transactions American Geophysical Union. 2008;89(10):93-4. doi: 10.1029/2008eo100001.

9. Qiu J, Turner MG. Spatial interactions among ecosystem services in an urbanizing agricultural watershed. Proceedings of the National Academy of Sciences. 2013;110(29):12149-54. doi: 10.1073/pnas.1310539110.

10. Panagos P, Van Liedekerke M, Montarella L. The European Soil Database distribution version 2.0; 2006. Accessed: <http://eusoils.jrc.ec.europa.eu/esdb_archive/ESDB/Index.htm>.

11. LfULG. Auswertekarten Bodenschutz 1:50.000; 2012. Accessed: <http://www.umwelt.sachsen.de/umwelt/boden/26192.htm>.

12. Bischoff R. K-Faktor (MMK 100); 2014. Accessed: <http://www.umwelt.sachsen.de/umwelt/boden/27787.htm>.

13. Jäckel G, Zink M, Marx A. Aufbereitung von gemessenen und simulierten Klimadaten; 2012.

14. FAO Geonetwork. Global map of monthly reference evapotranspiration - 10 arc minutes (GeoLayer). 2014. Available: <http://www.fao.org/geonetwork/srv/en/main.home>. Accessed November 04 2015.

15. Bräunig A. Dokumentation zur Berechnung und Ableitung R-Faktor Sachsen; 2013.
