# Supplementary material for: Assessing Regional-Scale Impacts of Short Rotation Coppices on Ecosystem Services by Modeling Land-Use Decisions
Source: PLoS One. 2016 Apr 15;11(4):e0153862. doi: 10.1371/journal.pone.0153862 (PMC4833342; doi:10.1371/journal.pone.0153862)
Supplement: S1 Text — This document contains the full description of the economic simulation model following the ODD+D protocol [40]. (DOCX) [file pone.0153862.s003.docx]

# S1 Text: full model description of economic simulation model

This document contains the full description of the spatially explicit economic simulation model. The model is an extended version of the model described in [Weise [1](#_ENREF_1)]. Here, the model description follows the ODD+D protocol [[2](#_ENREF_2)]. As an extension of the widely used ODD protocol [[3](#_ENREF_3), [4](#_ENREF_4)], the ODD+D protocol puts particular focus on the presentation of human decision-making.

# Purpose

The model has been developed to understand the determinants and impacts of the expansion of short rotation coppices (SRCs). The aim of the presented study was to apply the model to the Mulde watershed in Central Germany and to analyse impacts of SRCs on multiple ecosystem services (ESS) and biodiversity.

# Entities, state variables and scales

The model contains following entities: individual land users (from here on called agents), the landscape, grids cells as spatial units and economic markets of different agricultural products as institutions. Table A gives an overview on model entities and associated parameters and state variables.

Table A. Entities, parameters and state variables of the model

| **Entity** | **Parameters** | **State variables** |
| --- | --- | --- |
| Agent | Coordinates | Profit |
|  | Discount rate |  |
|  |  |  |
| Landscape | Size | Shares of land use types |
| Grid cell | Coordinates | Land use |
|  | Soil quality |  |
|  | Distance to CHP plants |  |
| Market for annual agricultural crops | Demand | Price |
|  |  | Supply |
| Market for SRC | Demand | Price |
|  |  | Supply |

Exogenous drivers of land use decisions, which are not influenced by processes during a model run, are: soil qualities, demands, number and location of combined heat and power (CHP) plants. Space is explicitly considered in the model. Each cell is occupied by one agent who stays in that cell for the whole simulation and decides in each time step on the land use type in that cell. One time step represents one year and simulations were run for 50 years.

# Process overview and scheduling

Here, the processes of the model are briefly specified to allow a general overview of the model and its dynamics. For a detailed description of the processes see section 8.

**Process 1: Initialization of landscape**

Before the start of the simulation, the landscape is initialized based on empirical spatial data, i.e., distribution of soil quality and spatial allocation of CHP plants.

**Process 2: Decision-making of agents**

In each time step all agents decide sequentially in a random order between three different land use types: no cultivation, annual agricultural crops for food or feed production, and SRCs.

**Process 3: Calculation of regional supply and market prices**

After each agent decision, the market prices for the different commodities (woody products from SRCs and other agricultural crops from annual cultivation) is updated based on the exogenously given demands and current regional supply.

# Design concepts

## Theoretical and empirical background

A main concept is the implementation of markets for the different agricultural commodities (woody products from SRCs and other agricultural crops from annual cultivation). Market prices in the model are determined by the balance of supply and demand. This price formation on the market is in line with standard economic theory (e.g., equilibrium concept; cf. [[5](#_ENREF_5), [6](#_ENREF_6)]). At this, market price is determined by an externally given demand and a supply that is solely generated by the land use decisions of the agents (i.e., endogenous markets). We assume that the market price is equivalent across all CHP plants and is determined by the joint supply from all agents (termed “regional price”). Besides utilizing SRC products, CHP plants are also distributing resources: plants with a high supply (i.e., higher than their own capacity) are able to transport and to sell to another plant or other customers. This means that the agents always supply the closest CHP plant as they receive the same price at each plant and the transport costs are minimal for the closest plant.

However, we tested this scenario against a second scenario in which the market price is formed separately for each of the CHP plants and is determined by the amount of SRC that is sold to this specific plant (termed “local price”). Here, market prices might vary between plants. In the current study, simulated land-use patterns and the regional ESS showed to be the equivalent across both price scenarios (see Table A and B in S1 File). Therefore, we focus on the “regional price” scenario in this study and will present the details of the “local price” scenario only verbally in the submodels below.

The agents are rational profit maximizers, i.e., agents have clear preferences over all possible land use options and aim at maximizing their income. At this, it is assumed that agents have full information (that are available in the model) and the needed cognitive ability to process all possible options. This decision model was chosen to be close to established theory. Furthermore, we believe that profit maximization is an appropriate assumption for industrial agricultural decisions. To enable the comparison between land use types with different lifespans, the equivalent annual annuity approach (see for example [Brigham and Houston [7](#_ENREF_7)]) from investment theory was chosen. This approach is appropriate as it is often recommended to land users interested in SRC practice in Germany (for example [Schweinle and Franke [8](#_ENREF_8)]) and has been used in several studies on the financial analysis of SRCs [[9](#_ENREF_9)].

## Individual decision-making

The agents, namely individual farmers, decide between three different land use types: no cultivation, annual crops for food or feed production and SRCs. Agents follow a rational profit maximization approach using an equivalent annual annuity approach (see above). Agents adapt to changing market prices. Neither social norms nor cultural values are incorporated in the model. Spatial aspects are incorporated as the distance to CHP plants influences the decision via resulting transportation costs. Temporal aspects play a role as discounting of future profits is incorporated in the equivalent annual annuity approach. Discount rates are seen as subjective discount rates which can vary depending on personal risk aversion [[10](#_ENREF_10)].

## Learning

Learning is not incorporated in the model.

## Individual sensing

The agent knows its current land use, the location factors of its land, i.e., soil quality and distance to CHP plants, and the current market price. The agents do not perceive information from other agents directly. However, they do sense the current total supply which comprises all agents currently chosen land use type. The sensing process is not modelled explicitly, it is not erroneous and no costs of sensing are incorporated.

## Individual prediction

Agents are not able to predict changes in market prices. However, they are able to predict how their own decision will impact the current market price.

## Interaction

Agents interact indirectly via the endogenous market. The land use decision of an agent influences the market prices, which then influences the land use decisions of other agents.

## Collectives

There are no collectives incorporated in the model.

## Heterogeneity

Agents are heterogeneous with regard to the location factors of their land. Here, the soil qualities as well as the distances to CHP plants differ between cells. Soil quality influences productivity of annual crops as well as of SRCs. Distances to CHP plants determine transportation costs of SRC products.

## Stochasticity

Agents make their decisions in a random order.

## Observation

With the goal of this study to assess impacts of SRC expansion in the Mulde watershed in Central Germany, the number and coordinates of SRCs are observed as main output variables.

# Implementation details

The model was implemented in C++ using Embarcadero® C++Builder® 2010.

# Initialization

The model landscape is initialized with spatial data on soil quality [[11](#_ENREF_11)] and CHP plants [[12](#_ENREF_12)]. At the beginning, all cells are not under cultivation.

# Input

Spatial data on soil quality [[11](#_ENREF_11)], CHP plants [[12](#_ENREF_12)] and on current land use [[13](#_ENREF_13), [14](#_ENREF_14)] is used.

# Submodels

## Initialization of landscape

Based on the current land use [[13](#_ENREF_13), [14](#_ENREF_14)], each cropland cell of the Mulde watershed is assigned to one agent. In total, this amounts to 9537 agents/cells. For each cell the soil quality value and the distances to the CHP plants are set based on the spatial data [[11](#_ENREF_11), [12](#_ENREF_12)]. For the “regional price” scenario, only the distance to the closest CHP plant is needed as the market price is equivalent across all CHP plants (see section 4.1); hence, agents always supply the closest CHP plant.

## Decision-making of agents

The agent chooses between three land use types: SRCs, annual agricultural crops (ANN) or no cultivation (NoC). From these, the agent chooses the option that maximizes its profits. The profit calculation differs between the three land use types. No cultivation yields neither costs nor revenue and its profit for agent *i* is therefore:

$P_{NoC}^{i}=0$

1

For annual agricultural crops the following profit function applies:

$P_{ANN}^{i}\left( t \right)= p_{ANN}\left( t \right)*{prod}_{ANN}^{i}-pca$

2

where $p_{ANN}\left( t \right)$ is the current market price (calculated by equation 10), ${prod}_{ANN}^{i}$ the productivity of annuals crops in the cell of agent $i$ and $pca$ the production costs of annuals. The productivity of annual crops is determined by the location factor soil quality by assuming a linear relationship:

${prod}_{ANN}^{i}={sq}^{i}$

3

where ${sq}^{i}$ is the soil quality of the cell of agent $i$. As pointed out by [Zhang *et al.* [15](#_ENREF_15)] soil properties strongly impact the agricultural output. Similarly, we assume that productivity and soil quality are linearly correlated with both factors being normalized between 0 and 1. . Thereby, we follow the concept of using soil values (“Bodenwertzahl”) to classify German soils [[16](#_ENREF_16)]. The soil value is a measure for differences in net yield under proper cultivation that are solely determined by differences in soil [[16](#_ENREF_16)]. With equation 3, a soil value ${sq}^{i}$ of 0.5 represents a reduction in net yield by 50% of the maximal yield [[15](#_ENREF_15), [17](#_ENREF_17)].

As SRCs represent long-term investment decisions, concepts of intertemporal choice should be taken into account in the profit calculation. The underlying idea is that people value profit differently at different points in time. For this study, the equivalent annual annuity approach (for example described in [Brigham and Houston [7](#_ENREF_7)]) from investment theory was chosen. This approach is appropriate as it is often recommended to land users interested in SRC practice in Germany (for example [Schweinle and Franke [8](#_ENREF_8)]) and has been used in several studies on the financial analysis of SRCs [[9](#_ENREF_9)].

In a first step, the profit of agent *i* in year *t* $P_{SRC}^{i}(t)$ over the whole life time of the SRC is calculated by:

$P_{SRC}^{i}\left( t \right)= \left\{ \begin{aligned} p_{SRC}\left( t \right)*{prod}_{SRC}^{i}*rot- {costs}_{i}(t)\text{, if} t mod rot=0 \\ - {costs}_{i}(t)\text{, } \text{else} \end{aligned} \right.$

4

where $p_{SRC}\left( t \right)$ is the current market price in year $t$ for SRC products produced in one year on optimal soil conditions calculated by equation 10, ${{prod}_{SRC}^{i}}$ the productivity of SRCs in the cell of agent $i$, *rot* the number of years after which SRCs are harvested and ${costs}_{i}\left( t \right)$ are all incurring costs in year $t.$ For the “regional price” scenario, this profit is only calculated for supplying the closest CHP plant because agents receive the same price at each plant and the transport costs are minimal for the closest plant. In contrast, for the “local price” scenario the profit differs between the 15 CHP plants present in the Mulde watershed because market prices $p_{SRC}\left( t \right)$ and transportation costs (included in the ${costs}_{i}\left( t \right)$ calculated by equation 6) are different between the 15 plants. These differences are more closely described below.

The productivity of SRCs is given by:

${{prod}_{SRC}^{i}}= \left\{ \begin{aligned} {prod}_{min}+0.2\text{, if} {sq}^{i}\geq0.5 \\ {prod}_{min}\text{, if} {sq}^{i}<0.5 \end{aligned} \right.$

5

where ${prod}_{min}$ is the productivity on cells with a soil quality ${sq}^{i}$ below 0.5. Hence, the productivity of SRCs is assumed to decrease on poor soils (as was found by [Ali [18](#_ENREF_18)]). At this, the dependence on soil quality is less pronounced than for annual crops (see equation 3) because studies showed that biomass yield from SRCs is dependent on further factors such as age of plantation or precipitation [[18](#_ENREF_18)]. Nevertheless, in our model the consideration of biophysical location factors is restricted to the soil quality due to simplicity reasons.

Finally, all occurring costs for agent *i* are calculated by:

${costs}_{i}=\left\{ \begin{aligned} ic, \text{if} t=0 \\ hc+rot*{tc}^{i}, \text{if} t mod rot=0 \text{and} t<LT \\ hc+rot*{tc}^{i}+rc, \text{if} t=LT \\ 0, else \end{aligned} \right.$

6

where $rot$ is the number years after which SRCs are harvested, $ic$ are the investment costs, $hc$ the harvest costs, ${tc}^{i}$ the transportation costs of wood produced per year and $rc$ the recovery costs. In the initial year the investment costs $ic$ are due, at the end of each rotation cycle harvest costs $hc$ as well as transportation costs to the CHP plant ${tc}^{i}$ occur and finally at the end of the lifetime additional recovery costs of the land $rc$ have to be paid. The transportation costs are linearly dependent on the distance to CHP plant:

${tc}^{i}={(tc}_{min}+{tc}_{slope}*d^{i})*{prod}_{SRC}^{i}*yield$

7

where $d^{i}$ is the distance of agent *i* to the closest CHP plant and calculated as Euclidean distance [[19](#_ENREF_19)] from the data on CHP plants [[12](#_ENREF_12)], ${tc}_{min}$ are fixed costs for transportation, ${tc}_{slope}$ the transport price per distance, ${prod}_{SRC}^{i}$ the productivity of SRCs in the cell of agent $i$ and $yield$ is the yield of SRC products produced in one year on optimal soil conditions. For the “regional price” scenario the distance $d^{i}$ is the distance to the closest CHP plant. For the “local price” scenario the transport costs to each of the 15 CHP plants in the Mulde watershed need to be calculated by equation 7 with $d^{i}$ being the distance to the specific CHP plant.

From the sequence of profits $P_{SRC}^{i}(t)$, the net present value is calculated as the sum of the discounted profits:

${NPV}^{i}= \sum_{t=0}^{LT} \left( 1+r \right)^{-t}*P_{SRC}^{i}(t)$

8

where $LT$ is the lifetime of the plantation, $r$ the discount rate and $P_{SRC}^{i}(t)$ the profit in year $t$ calculated by equation 4.

Subsequently, the equivalent annual value $EAV$ is calculated from the net present value $NPV$ to enable the comparison of land use options with unequal lifespans:

${EAV}^{i}= \frac{1}{1-{(1+r)}^{-LT}}*{NPV}^{i}$

9

where $r$ is the discount rate, $LT$ the lifetime of a SRC plantation and ${NPV}^{i}$ the net present value calculated by equation 8.

In a final step, the agent compares the equivalent annual value ${EAV}^{i}$ with the possible profit from annual agricultural production $P_{ANN}^{i}\left( t \right)$ and chooses the option with the higher profit. If both, the equivalent annual value ${EAV}^{i}$ of SRC and the profit of annual agricultural crops $P_{ANN}^{i}\left( t \right)$ would yield negative incomes, the agent decides to not cultivate its land in the current year.

## Calculation of regional supply and market prices

After each decision step, the regional supplies $S_{j}\left( t \right)$ and the market prices $p_{j}\left( t \right)$for the different products *j*, i.e., $ANN$ and $SRC$, are updated by calculating:

$p_{j}\left( t \right)= \frac{D_{j}}{S_{j}\left( t \right)}\text{with} S_{j}\left( t \right)= \sum_{i=1}^{N} h_{j}^{i}(t)$

10

where $D_{j}$ is the demand for product $j\in\left\{ ANN,SRC \right\}$, $N$ the number of agents and $h_{j}^{i}(t)$ the harvest amount of product $j$ in cell $i$ given by:

$h_{ANN}^{i}\left( t \right)= \left\{ \begin{aligned} {prod}_{ANN}^{i}\text{, if land use is ANN} \\ 0\text{, if land use is not ANN} \end{aligned} \right.$

11

$h_{SRC}^{i}\left( t \right)= \left\{ \begin{aligned} {prod}_{SRC}^{i}\text{, if land use is SRC} \\ 0\text{, if land use is not SRC} \end{aligned} \right.$

12

For the “local price” scenario, the market price $p_{j}\left( t \right)$ needs to be calculated separately for each of the 15 CHP plants. In that case the total demand $D_{SRC}$ is equally distributed between the 15 CHP plants and for the supply $S_{j}\left( t \right)$ only that of the specific CHP plant is taken.

# Parameter set

Table B shows the names of all parameters used in the model, their values and, if available, the references for their parameterization.

Table B. Parameters of the model

| **Parameter** | **Symbol** | **Value** | **Unit** | **Reference** |
| --- | --- | --- | --- | --- |
| **Technical parameters** | | | | |
| Number of agents | $N$ | 9537 | - | - |
| Number of time steps | $T$ | 50 | years | - |
| **Agent** | | | | |
| Discount rate | $r$ | 6% | - | average value of discount rates used in SRC studies included in a review by  [Kasmioui and Ceulemans [9](#_ENREF_9)] |
| **Annual crops** | | | | |
| Demand for annual crops | $D_{ANN}$ | 31000 | money units per year and ha | chosen based on initial shares of agricultural and fallow land currently present in case study [[13](#_ENREF_13), [14](#_ENREF_14), [20](#_ENREF_20)] |
| Production costs per ha | $pca$ | 2.4 | money units per ha | [Landwirtschaftskammer Niedersachsen [21](#_ENREF_21)] |
| **SRCs** | | | | |
| Demand for SRC products | $D_{SRC}$ | 460 | money units per year and ha | chosen based on current demand in case study (solely given by CHP plants present) [[12](#_ENREF_12)] |
| Investment costs per ha | $ic$ | 4.7 | money units | [Schweinle and Franke [8](#_ENREF_8)], [Wagner *et al.* [22](#_ENREF_22)] |
| Recovery costs per ha | $rc$ | 3.6 | money units per ha | [Schweinle and Franke [8](#_ENREF_8)], [Wagner *et al.* [22](#_ENREF_22)] |
| Harvest costs per ha | $hc$ | 1.6 | money unit per ha | [Schweinle and Franke [8](#_ENREF_8)], [Wagner *et al.* [22](#_ENREF_22)] |
| Rotation cycle | $rot$ | 4 | years | [Aylott *et al.* [23](#_ENREF_23)], [Hillier *et al.* [24](#_ENREF_24)] |
| Lifetime | $LT$ | 20 | years | maximal number of years to not count as forest [[25](#_ENREF_25)] |
| Minimal productivity | ${prod}_{min}$ | 0.8 | - | - |
| Minimal transportation costs per dry ton | ${tc}_{min}$ | 0.02 | money units per dry ton | linear regression based on values from  [Kröber *et al.* [26](#_ENREF_26)], [Strohm *et al.* [27](#_ENREF_27)], [Wagner *et al.* [22](#_ENREF_22)] |
| Slope transportation costs | ${tc}_{slope}$ | 0.001 | money units per dry ton and distance | linear regression based on values from  [Kröber *et al.* [26](#_ENREF_26)], [Strohm *et al.* [27](#_ENREF_27)], [Wagner *et al.* [22](#_ENREF_22)] |
| Yield per ha | $yield$ | 12 | dry tons per ha | [Aust *et al.* [28](#_ENREF_28)] |

# References

1. Weise H. Land use change in the context of bioenergy production: impact assessment using agent-based modelling [PhD Thesis]: University of Osnabrück; 2014.

2. Müller B, Bohn F, Dreßler G, Groeneveld J, Klassert C, Martin R, et al. Describing human decisions in agent-based models – ODD + D, an extension of the ODD protocol. Environmental Modelling & Software. 2013;48:37-48.

3. Grimm V, Berger U, Bastiansen F, Eliassen S, Ginot V, Giske J, et al. A standard protocol for describing individual-based and agent-based models. Ecological Modelling. 2006;198(1–2):115-26.

4. Grimm V, Berger U, DeAngelis DL, Polhill JG, Giske J, Railsback SF. The ODD protocol: A review and first update. Ecological Modelling. 2010;221(23):2760-8.

5. Mankiw NG, Taylor MP. Economics: Thomson Learning Services, Toronto; 2006.

6. Engelkamp P, Sell F. Einführung in die Volkswirtschaftslehre: Springer, Berlin, Heidelberg; 2007.

7. Brigham E, Houston J. Fundamentals of Financial Management: Cengage Learning; 2006.

8. Schweinle J, Franke E. Beratunsghandbuch zu Kurzumtriebsplantagen. In: Skodawessely PB, editor.: Eigenverlag der TU Dresden; 2010.

9. Kasmioui OE, Ceulemans R. Financial analysis of the cultivation of poplar and willow for bioenergy. Biomass and Bioenergy. 2012;43(0):52-64.

10. Barberis N, Thaler R. Handbook of the Economics of Finance. Constantinides GM, Harris M, Stulz R, editors. Elsevier Science; 2003.

11. LfULG. Auswertekarten Bodenschutz 1:50.000; 2012. Accessed: <http://www.umwelt.sachsen.de/umwelt/boden/26192.htm>.

12. Das S, Eichhorn M, Hopffgarten MV, Lang E, Priess J, Thrän D. Spatial Analysis of the Potential of District Heating from Existing Bioenergy Installations in Germany; 2012; Milan. ETA-Florence Renewable Energies.

13. Wochele S, Priess J, Thrän D, O’Keeffe S. Crop allocation model “CRAM” - an approach for dealing with biomass supply from arable land as part of a life cycle inventory. In: Hoffmann C, Baxter, D., Maniatis, K., Grassi, A., Helm, P., editor; 2014; Hamburg. ETA-Florence Renewable Energies.

14. Wochele-Marx S, Lang E, Pomm S, Das S, Priess J. Central Germany GIS dataset; 2015. Database: figshare. Accessed: https://figshare.com/articles/Central_Germany_GIS_dataset/1318765/2.

15. Zhang W, Ricketts TH, Kremen C, Carney K, Swinton SM. Ecosystem services and dis-services to agriculture. Ecol Econ. 2007;64(2):253-60. doi: 10.1016/j.ecolecon.2007.02.024. PubMed PMID: WOS:000252264600003.

16. GD NRW. Wertzahlen der Bodenschätzung. 2014. Available: [www.gd.nrw.de](http://www.gd.nrw.de). Accessed April 15 2015.

17. Petzold R, Butler-Manning D, Feldwisch N, Glaser T, Schmidt PA, Denner M, et al. Linking biomass production in short rotation coppice with soil protection and nature conservation. Iforest. 2014;7:353-62. doi: 10.3832/Ifor1168-007. PubMed PMID: WOS:000344960400002.

18. Ali W. Modelling of Biomass Production Potential of Poplar in Short Rotation Plantations on Agricultural Lands of Saxony, Germany [PhD Thesis]: Dresden University of Technology; 2009.

19. Deza MM, Deza E. Encyclopedia of Distances: Springer, Berlin, Heidelberg; 2013.

20. European Environment Agency (EEA). Corine Land Cover 2006 raster data; 2013. Accessed: <http://www.eea.europa.eu/data-and-maps/data/corine-land-cover-2006-raster-3>.

21. Landwirtschaftskammer Niedersachsen. Maispreisrechner. 2014. Available: <http://www.lwk-niedersachsen.de/index.cfm/portal/betriebumwelt/nav/360/article/17878.html>. Accessed April 16 2015.

22. Wagner P, Schweinle J, Setzer F, Kröber M, Dawid M. DLG-Standard zur Kalkulation einer Kurzumtriebsplantage. 2012.

23. Aylott MJ, Casella E, Tubby I, Street NR, Smith P, Taylor G. Yield and spatial supply of bioenergy poplar and willow short-rotation coppice in the UK. New Phytologist. 2008;178:358–70.

24. Hillier J, Whittaker C, Dailey G, Aylott M, Casella E, Richter G, et al. Greenhouse gas emissions from four bioenergy crops in England and Wales: Integrating spatial estimates of yield and soil carbon balance in life cycle analyses. GCB Bioenergy. 2009;1(4):267-81.

25. Gesetz zur Erhaltung des Waldes und der Förderung der Forstwirtschaft (BWldG). 2010. Bundesministerium für Justiz und Verbraucher.

26. Kröber M, Becker R, Reike J, Wolf H. Transport. In: Skodawessely PB, editor. Beratunsghandbuch zu Kurzumtriebsplantagen: Eigenverlag der TU Dresden; 2010.

27. Strohm K, Schweinle J, Liesebach M, Osterburg B, Rödl A, Baum S, et al. Kurzumtriebsplantagen aus ökologischer und ökonomischer Sicht. 2012.

28. Aust C, Schweier J, Brodbeck F, Sauter UH, Becker G, Schnitzler JP. Land availability and potential biomass production with poplar and willow short rotation coppices in Germany. GCB Bioenergy. 2014;6(5):521-33.
